# Exploring the Nutraceutical Potential of a Food–Medicine Compound for Metabolic-Associated Fatty Liver Disease via Lipidomics and Network Pharmacology

**DOI:** 10.3390/foods14071257

**Published:** 2025-04-03

**Authors:** Yuru Deng, Jie Cui, Yuxuan Jiang, Jian Zhang, Jinchi Jiang, Quanbin Zhang, Yonghong Hu

**Affiliations:** 1 College of Food Science and Light Industry, Nanjing Tech University, Nanjing 211816, China; dengyuru-@njtech.edu.cn (Y.D.); four-leaf-clover@njtech.edu.cn (J.Z.); jiangjinchi@126.com (J.J.); yonghonghuyg@163.com (Y.H.); 2 Institute of Oceanology, Chinese Academy of Sciences, Qingdao 266071, China; qbzhang@qdio.ac.cn; 3 College of Biotechnology and Pharmaceutical Engineering, Nanjing Tech University, Nanjing 211816, China; jyxxxx@njtech.edu.cn

**Keywords:** metabolic-associated fatty liver disease, food and medicine homology, natural products, network pharmacology, lipidomics

## Abstract

Metabolic-associated fatty liver disease (MAFLD) is a prevalent global health issue closely tied to dietary habits, impacting a significant portion of the adult population. MAFLD is linked to various metabolic disorders, elevating risks of cirrhosis and hepatocellular carcinoma and severely impacting patients’ quality of life. While therapeutic research has progressed, effective food-based interventions remain scarce. Natural products, rich in bioactive compounds and offering health benefits, have gained attention for their potential in managing MAFLD. This study employed network pharmacology and lipidomics to investigate the therapeutic effects of Food and Medicine Homology (FMH) on MAFLD using a high-fat-diet-induced HepG2 cell model. We identified 169 potential bioactive components from *Radix Puerariae*, *Hericium erinaceus*, *Rhizoma Curcumae longae*, *Camellia oleifera*, and *Hoveniae Dulcis* Semen, constructing a drug–component–target network that highlighted 34 key targets. The characteristic components of this FMH compound solution (HSD) were identified using UPLC-QTOF-MS/MS. In vitro, HSD significantly reduced intracellular lipid accumulation, decreased inflammatory markers, and mitigated hepatocyte damage. Lipidomics analysis revealed significant alterations in lipid metabolites, suggesting HSD’s potential to modulate sphingolipid and glycerophospholipid metabolism, thus improving MAFLD outcomes. This research underscores the critical role of the FMH complex in modulating lipid metabolism and inflammatory pathways, offering valuable insights for developing FMH-based dietary supplements and functional foods to alleviate MAFLD. By leveraging the synergistic effects of natural compounds, our findings hold significant implications for innovative nutritional strategies in managing this prevalent metabolic disorder.

## 1. Introduction

Metabolic-associated fatty liver disease (MAFLD), previously known as non-alcoholic fatty liver disease, has become one of the most prevalent chronic liver diseases, impacting approximately 25.24% of the global adult population [1]. This condition encompasses a spectrum of severity, ranging from simple steatosis to steatohepatitis and fibrosis, and poses a significant risk for the development of cirrhosis and hepatocellular carcinoma [2], thereby adversely affecting patients’ quality of life. Economically, MAFLD imposes a substantial burden on healthcare systems, with annual direct medical costs in the US estimated to be around USD 1030 per patient [3]. The interplay of obesity, diabetes, and aging is expected to escalate MAFLD-related liver diseases and mortality, with projections indicating over 80,000 liver deaths between 2015 and 2030 [4].

Despite significant advancements in drug development, there are currently no medications approved by the Federal Drug Administration (FDA) for the treatment of MAFLD [5]. In the absence of effective pharmacological options, lifestyle interventions, such as increased physical activity, caloric restriction, and strategies, to reduce hepatic lipid levels and enhance insulin sensitivity are crucial. Numerous studies have demonstrated the efficacy of FMH in preventing or ameliorating MAFLD [6], highlighting its potential as a novel therapeutic approach.

Nutrition is the cornerstone of human health and vitality. Among the diverse categories of edible substances, those with inherent medicinal properties, known as Food–Medicine Homology (FMH), hold a unique and significant place in dietary culture. As our understanding of these dual-purpose substances deepens, they are increasingly being incorporated into modern healthcare, supplementing traditional treatments, enhancing the immune system, and gradually being used as adjuncts in disease mitigation and therapy [7]. FMH is valued for its rich nutritional and medicinal benefits, which are closely linked to its unique array of natural compounds and their concentrations. Many FMHs, such as ginseng [8], aloin, licorice, *Puerariae Flos* [9], and goji berries, are natural antioxidants that can mitigate reactive oxygen species accumulation and protect hepatocytes, thereby preventing the onset and progression of MAFLD [6]. Several FMHs, including Simao, Gegen Qinlian Decoction, Huanglian Jiedu Decoction, and LLKL, have shown potential in treating MAFLD by altering gut microbiota composition and influencing their metabolic activities [10]. Additionally, the Xiao–Ke–Yin formulation, composed of nine FMHs, has been shown to improve MAFLD by downregulating genes in the liver cholesterol biosynthesis pathway and modulating the dysbiosis of the gut microbiota and its metabolites [11]. Unlike single-target drugs, FMH encompasses multiple bioactive components that work synergistically through multi-target and multi-pathway mechanisms, offering a more comprehensive approach to improving liver health. Its anti-inflammatory and antioxidant properties, along with its ability to regulate lipid metabolism and insulin sensitivity, contribute to its holistic therapeutic effects. In contrast, conventional drugs, like metformin and vitamin E, may cause gastrointestinal discomfort or increase bleeding risk. FMH, sourced from common foods, exhibits superior safety and tolerability, with minimal side effects, even during extended use. For instance, the regular consumption of teas such as *Ampelopsis grossedentata* [12], Anhua fuzhuan tea [13], and *Gynostemma pentaphyllum* [14] has demonstrated beneficial effects, including lipid and glucose regulation, as well as hepatoprotective properties. Furthermore, FMH can be tailored to individual needs, facilitating personalized treatment and emphasizing disease prevention through dietary and lifestyle modifications, thereby promoting long-term health. The judicious combination of FMH can enhance the beneficial effects of its natural bioactive compounds. With the growing awareness of health and wellness, the future growth potential of FMH-based food supplements in the high-end health food and functional food markets is substantial.

However, the inherent complexity of FMH compound solutions, which include a multitude of chemical components, necessitates extensive research and clinical experience, thereby limiting the development of FMH-based solutions [6]. The interactions of active substances in various FMHs increase the complexity of the components, and the effects on different individuals vary significantly due to factors such as genotype and gut microbiota. Consequently, the exploration of FMH for alleviating MAFLD is still in its nascent stages. By integrating network pharmacology analysis with FMH, it is possible to develop food-based nutritional strategies for MAFLD. Network pharmacology, an interdisciplinary approach at the forefront of systems biology, pharmacology, and bioinformatics, uncovers the molecular mechanisms and network-based drug-effect regulation [8,12,13,14], offering a novel perspective for investigating multi-component and multi-target therapeutics within FMH [15].

For the current study, we intend to harness network pharmacology to identify bioactive compounds and their targets from FMH compound solutions and food-like products with potential efficacy in alleviating MAFLD. Guided by the insights gleaned from network pharmacology analyses, we selected *Radix Puerariae*, *Hericium erinaceus*, *Rhizoma Curcumae longae* (*C. longa*), *Camellia oleifera*, and *Hoveniae Dulcis* Semen to formulate a novel FMH compound solution: “HepaSynergy Decoction” (HSD). Our objective is to ascertain the efficacy of HSD in mitigating cellular fat accumulation using a fatty acid-induced HepG2 cell. Furthermore, lipidomics analysis is implemented to elucidate the hepatoprotective mechanisms of HSD, providing a deeper understanding of its nutraceutical potential in MAFLD management.

## 2. Materials and Methods

### 2.1. Preparation of Crude Plant Extracts

The mixture of *R. puerariae*, *H. erinaceus*, *C. longa*, *C. oleifera*, and *H. Dulcis* Semen was acquired from Zhejiang Changfa Cereals Oils and Foods Co., Ltd. (Quzhou, China). Throughout the experimental process, we implemented standardized screening and collection procedures. The materials underwent controlled processing conditions, involving uniform drying at 60 °C and grinding to a particle size that passed through an 80-mesh sieve. The resulting powders were then combined in a specific ratio of 4:6:4:62:4 (m/m) to prepare the HSD at a concentration of 80 mg/mL. For subsequent experiments evaluating the effects on liver damage, the HSD was diluted using a two-fold gradient to prepare final concentrations of 1.25, 2.5, 5, 7.5, 10, 15, and 25 μL/mL.

### 2.2. Network Pharmacology Analysis of Potential Bioactive Compounds

Potential active compounds were initially identified using the HERB database [16] (http://herb.ac.cn/, accessed on 11 September 2024) and further supported by a comprehensive literature review. The SwissADME tool [17] was employed to assess gastrointestinal absorption and adherence to Lipinski’s rule of five [18], which mandates that a compound should have a molecular weight not exceeding 500, an octanol–water partition coefficient that remains below 5, no more than 10 hydrogen bond acceptors, and no more than 5 hydrogen bond donors, with at least three of these criteria being satisfied. This process served to refine the selection of active compounds. Following this, the Swiss Target Prediction database [19] (http://www.swisstargetprediction.ch) was leveraged to forecast potential targets, considering genes with probabilities above 0.1 as likely targets of the compounds. Duplicates among the targets and compounds lacking suitable targets were excluded.

Potential targets associated with MAFLD were retrieved from the Human Gene Database (GeneCards, https://www.genecards.org/), the DisGeNET Database (https://www.disgenet.org/home/), and the OMIM Database (https://omim.org/) by querying with the term “liver injury” [20].

The screened targets of the active components and MAFLD-related proteins were uploaded to a Venn diagram webtool (http://bioinformatics.psb.ugent.be/webtools/Venn/, accessed on 31 March 2025) for analysis, leading to the identification of common targets. After removing duplicates, a cumulative set of MAFLD-related targets was collated and a drug–component–target network was constructed using Cytoscape v.3.7.0.

To examine the interplay between the chosen active compounds and their target proteins, the STRING v.11.0 interaction database platform (https://string-db.org/) was used to build a Protein–Protein Interaction (PPI) network [21]. Subsequently, Cytoscape v.3.7.0 software was used to analyze the targets highlighted by STRING within the context of MAFLD-related proteins, facilitating the visualization and analysis of the intricate interaction network. The Cytoscape plug-in CentiScaPe was applied to identify pivotal targets within the PPI network (BC > 377.30, CC > 0.00186, DC > 18.355).

The principal biological processes linked to the genes were scrutinized and underwent enrichment analysis via the online platform Metascape (http://metascape.org/gp/index.html) [22]. The outcomes were visualized using online biological tools. An enrichment analysis of the Kyoto Encyclopedia of Genes and Genomes (KEGG) pathways was conducted using the Cytoscape plugin ClueGO. Candidate genes implicated in MAFLD-related genes targeted by the active compounds were entered into the ClueGO plugin, with a *p* < 0.01 and *κ* ≥ 0.53.

Utilizing the KEGG pathway enrichment analysis, potential MAFLD-related genes targeted by the selected active compounds were pinpointed. These targets were substantiated through molecular docking with experimentally validated active compounds. The crystalline structures of the authenticated compounds were retrieved from the RCSB Protein Data Bank (https://www.rcsb.org/) [23]. The compound structures were preserved in the MOL2 format as docking ligands. iGEMDOCK software 2020 was engaged for molecular docking, with the standard docking procedure operating with default parameters.

From the molecular docking outcomes, the top-five receptor proteins with the most favorable energy scores and the most stable ligand–receptor complexes were selected. Subsequent to this, docking simulations were conducted using AutoDock Vina v.1.2.0. For the visualization of the docked complexes, PyMOL v.3.0 software was utilized for network representation and construction. The predictions generated by network pharmacology must be validated through experimental methods to confirm their biological relevance. This includes in vitro and in vivo studies to assess the compound-target interactions and their functional outcomes. The predictions generated by network pharmacology must be validated through experimental methods to confirm their biological relevance. This includes in vitro and in vivo studies to assess the compound–target interactions and their functional outcomes.

### 2.3. UPLC-QTOF-MS/MS Conditions

The mixture of *R. puerariae*, *H. erinaceus*, *C. longa*, *C. oleifera*, and *H. Dulcis* Semen was ground and prepared in a ratio of 4:6:4:62:4 (m/m). An aliquot of 1.0 g of the mixed powder was dispersed in 40 mL of an 80% methanol aqueous solution (v/v) and subjected to ultrasonic treatment in a water bath at room temperature for 60 min. The supernatant was then filtered through a 0.22 μm membrane filter to prepare the analytical solution. The analysis was performed on a Waters CORTECS UPLC C18 column (2.1 mm × 150 mm, 1.6 μm). The mobile phase consisted of methanol (A) and a 0.1% formic acid aqueous solution (B) with the following gradient elution program: 0–1.5 min: 10–15% A; 1.5–5 min: 15–30% A; 5–16.5 min: 30–58% A; 16.5–24 min: 58–78% A; 24–30.5 min: 78–98% A; and 30.5–33 min: 98% A. The flow rate was set at 0.25 mL/min, the column temperature was maintained at 40 °C, and the injection volume was 1 μL. Mass spectrometry analysis was conducted using an Agilent 6545 series quadrupole time-of-flight mass spectrometer (QTOF MS) (Agilent Technologies, Santa Clara, CA, USA) in both positive and negative ion modes. The key parameters were set as follows: Capillary voltage: 3500 V; Nebulizer pressure: 45 psig; Drying gas flow rate: 8 L/min; Drying gas temperature: 325 °C; and Fragmentor voltage: 145 V. Data acquisition was controlled by MassHunter Workstation software (Agilent Technologies, Santa Clara, CA, USA) [24].

### 2.4. MAFLD In Vitro Cell Model

HepG2 cells were purchased from the American Type Culture Collection and grown in DMEM supplemented with 100 IU/mL penicillin, 0.1 mg/mL streptomycin, and 10% (v/v) heat-inactivated fetal bovine serum at 37 °C in a 5% CO_2_ atmosphere.

According to the literature [25], HepG2 cells were cultured in a 37 °C, 5% CO_2_ incubator for 24 h with 0.5 mM free fatty acids (oleic acid (OA, Merck O7501):palmitic acid (PA, Merck P0500) = 2:1). The experiment was divided into normal control group and model intervention group, with 2.5, 5, and 10 μL/mL HSD treatments, with 3 replicates per group. HepG2 cells were seeded at 1.5 × 10^4^ cells per well in a 6-well plate and allowed to grow to approximately 60% confluence. The control and model intervention groups were cultured with complete medium for 24 h, while the HSD intervention groups were cultured with the experimental groups for 24 h. After discarding the original medium, cells were washed with PBS twice, and the model groups were treated with medium containing 0.5 mM OA:PA = 2:1 for 24 h, while the control group continued to be cultured with fresh complete medium.

### 2.5. Cell Viability Analysis by CCK-8 Assays

HepG2 cells in the logarithmic growth phase were seeded into 96-well plates at a density of 2 × 10^4^ cells/well and incubated overnight at 37 °C in a humidified atmosphere containing 5% CO_2_. Upon reaching a confluence of approximately 50–70%, the culture medium was discarded. Cells were then distributed into various groups: control and HSD groups with different concentrations (0, 1.25, 2.5, 5, 7.5, 10, 15, 20, and 25 μL/mL). Except for the control group, 100 μL of cell suspension was added to each well of the 96-well plate and returned to the cell culture incubator at 37 °C with 5% CO_2_ for 24 h to allow the cells to adhere. Following adhesion, the medium was replaced with 100 μL of fresh medium in each well, with six replicates per group. After another 24 h incubation, a 10 μL CCK-8 assay solution (Merck 96992-500TESTS-F) was added to each well. Three hours later, absorbance was measured at 450 nm to assess cell viability and proliferation.

$$Cell\;viability=\frac{\left({OD}_{i}-{OD}_{c}\right)}{\left({OD}_{0}-{OD}_{c}\right)}\times 100\%$$

In this context, OD*_i_* represents the absorbance values at 450 nm for HSD groups with different concentrations, OD_0_ represents the absorbance values at 450 nm for the HSD groups with a concentration of 0 μL/mL, and OD*_c_* represents the absorbance values at 450 nm for the control group.

### 2.6. Observation of Intracellular Lipid Droplets by Oil Red O Staining

According to the staining method of Zeng Lu [26], cells were collected after stimulation, and the original medium was discarded. Cells were washed twice with PBS, fixed with 4% paraformaldehyde for 30 min, and the fixation solution was discarded, washed twice with distilled water, treated with 60% isopropanol for 5–10 min, and then stained with oil red O working solution for 30 min. Excessive oil red O solution was washed with 60% isopropanol, and the remaining dye was removed with distilled water. Cells were observed under an inverted microscope to evaluate lipid deposition.

### 2.7. Determination of Intracellular Lipid Related Indicators

After the treatment, the medium was discarded and the cells were washed twice with PBS. 2% Triton X-100 was added to a concentration of 98% and 80 μL/well was added to the 6-well plate to lyse the cells for 30 min. Lipid accumulation-related markers were determined using assay kits, including the nitric oxide assay kit (Beyotime Biotechnology S0021S), triglyceride (TG) assay kit (Merck MAK264), total cholesterol (TC) quantification assay kit (Merck CS0005), as well as the ALT assay kit (Merck MAK264) and AST assay kit (Merck MAK467).

### 2.8. Lipidomics Analysis

After the treatment, the medium was discarded and the cells were washed twice with PBS. The cell precipitate was centrifuged for 15 min, and the supernatant was discarded after washing with PBS twice. Chloroform:methanol (2:1 v/v) was added to extract the cells. The sample was dried with nitrogen gas and reconstituted with 200 μL isopropanol. Mobile phase A was 10 mM ammonium acetate in water:acetonitrile (6:4, v/v) and mobile phase B was 10 mM ammonium acetate in isopropanol:acetonitrile (9:1, v/v). The gradient ranged from 40% B to 100% B (0–10 min), 100% B (10–12 min), and then held at 40% B to 15 min. The flow rate was set to 0.4 mL/min, and the column was a Zorbax Eclipse Plus C18 RRHD 2.1 × 50 mm, 1.8 μm, with a column temperature of 55 °C and an injection volume of 5.00 μL at 4 °C.

### 2.9. Statistical Analysis

Each major experiment was performed at least three times. Statistical analysis was performed using SPSS 17.0, and all the results are expressed as the mean ± standard deviation. Comparisons among groups were performed using one-way ANOVA followed by Tukey’s multiple comparison.

## 3. Results

### 3.1. Prediction of Potential Active Ingredients and Key Targets for Repairing Liver Injury Based on Network Pharmacology

In our initial screening, we identified 355 potential active components, which were further refined to 169 potential active compounds based on SwissADME and Lipinski’s rule of five. Among these, 75 compounds originated from *C. longae*, 52 from *H. erinaceus*, 17 from *R. puerariae*, 15 from *H. Dulcis* Semen, and 10 from *C. oleifera*. Notably, we identified several common components shared between *C. longa* and *H. erinaceus*, including Benzaldehyde (CID240), Phenylacetaldehyde (CID998), Linalool (CID6549), Decanal (CID8175), and L-Octanoylcarnitine (CID11953814).

**Figure 1:**
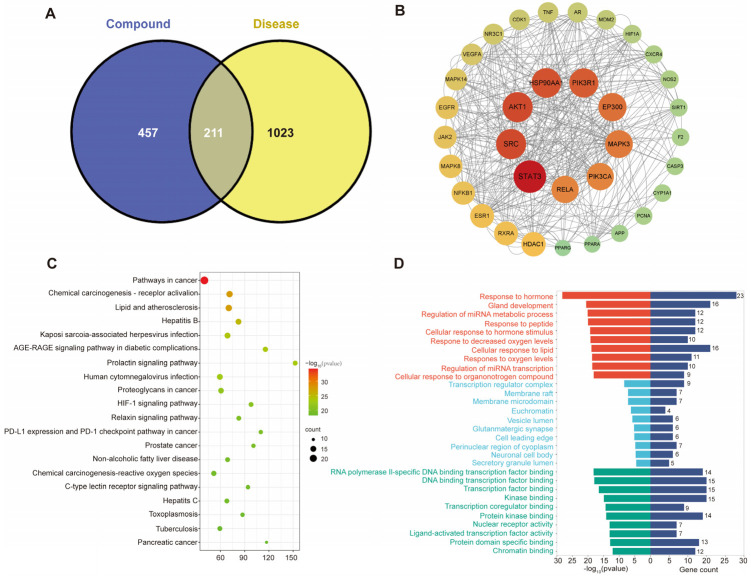
Network pharmacology analysis of potential active ingredients for alleviating metabolic-associated fatty liver disease (MAFLD). (**A**) Venn diagram of potential active ingredients and MAFLD-related targets involving the predicted bioactive compounds (in blue circle) and the disease targets (in yellow circle); (**B**) Protein–Protein Interaction network of the potential targets. Each node in the network represents a protein target, with larger and darker-colored nodes representing more significant targets; (**C**) bubble chart of the KEGG pathway. The horizontal axis represents the abundance factor and the vertical axis represents the pathway. The bubble size indicates the quantity of targets within each pathway. The bubble color signifies the *p*-value, with darker-red hues indicating a lower *p*-value; (**D**) histogram of GO enrichment analysis involving biological processes (red), cell components (blue), and molecular functions (green).

In the Swiss Target Prediction database, we predicted potential targets with a probability > 0.1 for the compounds, resulting in 668 targets. Using the keyword “liver injury”, we collected 1234 disease targets from the DisGeNet, GeneCards, and OMIM databases. The Venn diagram revealed 211 overlapping targets between the predicted bioactive compounds and the disease targets (Figure 1A). We then summarized and named the components associated with these targets, identifying 18 from *C. longae*, 12 from *H. erinaceus*, 9 from *R. puerariae*, 8 from *H. Dulcis* Semen, and 4 from *C. oleifera*. Decanal (CID8175) was a shared component between *C. longae* and *H. erinaceus* (Table S1). A drug–component–target network was constructed using Cytoscape (Figure S1). Through STRING analysis with a confidence score of 0.9, we obtained a network with 180 nodes and 1652 edges, after removing disconnected nodes. We then identified 34 key targets with BC > 377.30, CC > 0.00186, and DC > 18.355 (Figure 1B).

After performing the KEGG and GO enrichment analysis on the 34 key targets, we identified 20 pathways for further analysis (Figure 1C and Table S2). Among these, non-alcoholic steatosis, chemical carcinogenesis ROS, and AGE-RAGE signaling pathways in diabetic complications were directly related to steatosis. These pathways collectively promote an abnormal accumulation of fat in hepatocytes, ultimately leading to the occurrence and development of MAFLD. The GO enrichment results are shown in Figure 1D.

**Figure 2:**
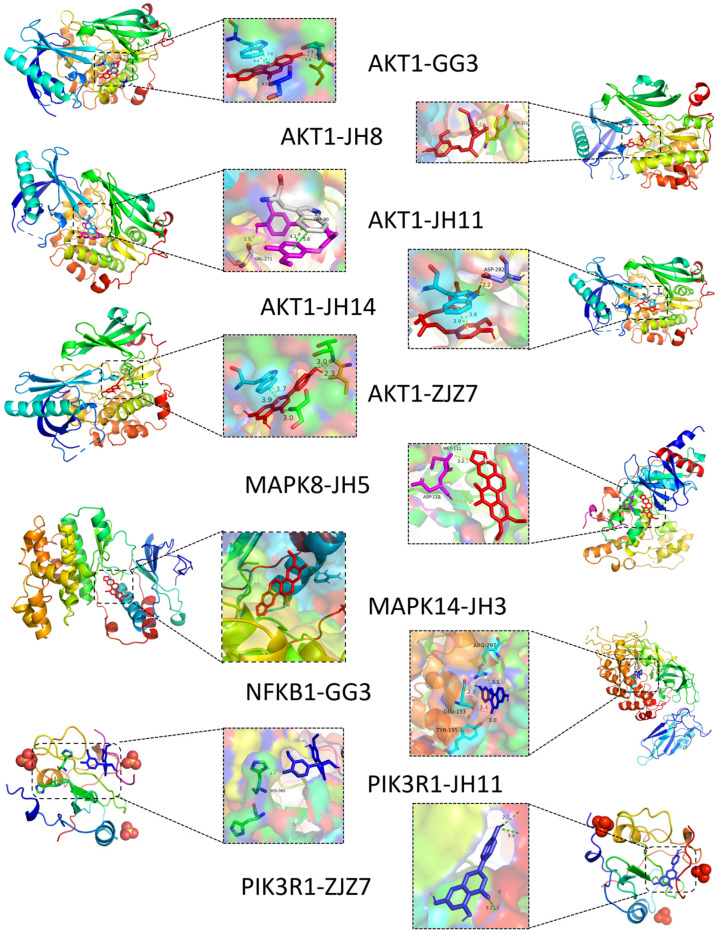
Protein interactions between seven potential active ingredients and five MAFLD-related key targets. GG3, coumestrol; JH3, nitidine; JH5, oxynitidine; JH8, curcumin; JH11, hexahydrocurcumin; JH14, (1Z,6Z)-1,7-bis(4-hydroxy-3-methoxyphenyl)hepta-1,6-diene-3,5-dione; ZJZ7, apigenin.

In the KEGG pathways with the highest correlation, we selected nine genes and their corresponding fifteen compounds for molecular docking analysis (Table S3). The binding energy threshold for significant interactions is generally considered to be less than −4.25 kcal/mol, with values less than −5.0 kcal/mol indicating good binding activity, and less than −7.0 kcal/mol indicating strong binding activity with high potential biological activity. The visualization analysis of ten pairs with a binding energy less than −7.0 kcal/mol are shown in Figure 2, and the chemical structures of these active ingredients are listed in Table S4. The results show that coumestrol, apigenin, (1Z,6Z)-1,7-bis(4-hydroxy-3-methoxyphenyl)hepta-1,6-diene-3,5-dione, hexahydrocurcumin, oxynitidine, and curcumin interact with five MAFLD-related key targets (AKT1, PIK3R1, MAPK8, MAPK14, and NFKB1). These compounds are spatially clustered, indicating strong interactions. Specifically, coumestrol, an isoflavonoid, is hypothesized to prevent hepatic steatosis through its cooperation with the GH signaling pathway both in vitro and in vivo [27]. Curcumin and its metabolic derivatives, hexahydrocurcumin and dihydrocurcumin, have been shown to inhibit the protein expression of RELA, PTGS2, IL-6, SRC, and AKT1, thereby exerting an anti-liver fibrosis effect [28]. Apigenin has been reported to reduce ethanol-induced inflammatory factors, such as NF-κB in hepatocytes [29]. These targets are implicated in pathways related to tumor development, chemical damage, and atherosclerosis. However, the full range of functions of these natural active ingredients requires further exploration.

**Figure 3:**
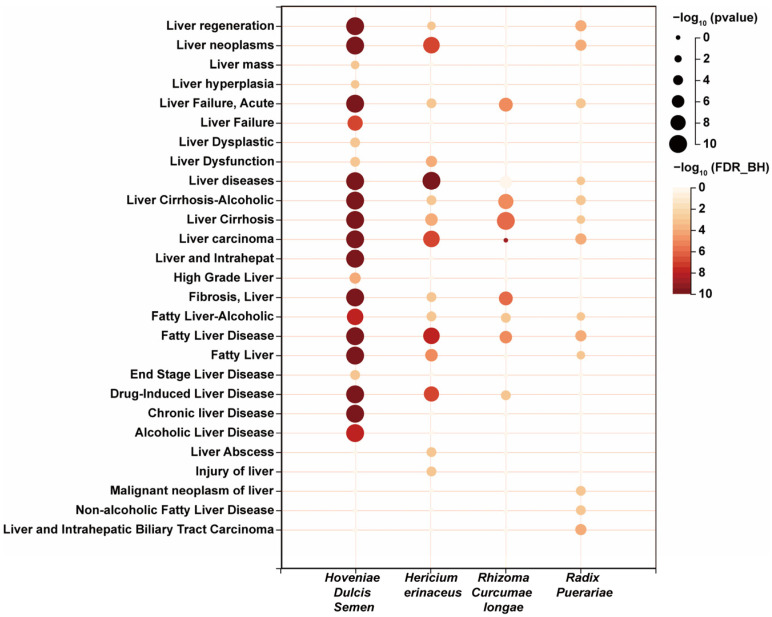
The HERB database offers substantiated evidence for the alleviation and therapeutic efficacy of liver disorders associated with the use of *Radix Puerariae*, *Hericium erinaceus*, *Rhizoma Curcumae longae*, and *Hoveniae Dulcis* Semen. Bubble size represents the significance level of the findings, with larger bubbles indicating a lower *p*-value. Bubble color indicates the false discovery rate adjusted using the Benjamini–Hochberg procedure, denoted as FDR_BH. Darker-red hues signify the higher reliability of the results.

*R. puerariae*, *H. erinaceus*, *C. longa*, *C. oleifera*, and *H. Dulcis* Semen were chosen to formulate a FMH compound solution through a systematic compounding process, named “HepaSynergy Decoction (HSD)”. The HERB database provides compelling evidence supporting the therapeutic benefits of these botanicals in the mitigation and management of hepatic conditions, as shown in Figure 3. Known for its hepatoprotective, lipid-lowering, anti-inflammatory, and alcohol-clearing properties, *H. dulcis* has been documented in 44 studies on platforms like Herb and TCMSP, with flavonoids constituting 27.61% of its main bioactive components [30]. Among these, nine chemical compounds—beta-sitosterol, dihydromyricetin, quercetin, naringenin, lutein, myricetin, kaempferol, emodin, and apigenin—are considered potential therapeutic agents for alcohol-related liver diseases [29,31,32,33,34,35,36,37]. *H. erinaceus* is a highly medicinal edible mushroom that has shown significant therapeutic potential for MAFLD through its effective protection of the gastric mucosa, anti-inflammatory effects, and regulation of lipid metabolism [38,39,40,41]. *C. longa* is often combined with other FMH substances to produce various nutritional benefits. Clinically, the combination of *C. longa* and turtle shell mainly exhibits hepatoprotective effects [42,43]. *R. puerariae* is commonly used as a key active ingredient in functional foods. With a relatively high content of isoflavones, *R. puerariae* has been shown to alleviate liver injury [44,45]. It offers a promising avenue within the integrative approach to liver health and disease management. It is imperative to acknowledge that the correlation between *C. oleifera* and MAFLD warrants further investigation.

Next, our research integrated network pharmacology with experimental validation. Using the Agilent 6545 QTOF Mass Spectrometer, we conducted metabolite identification for HSD. Through MS/MS analysis, we identified a total of 491 chemical components, with 197 detected in the negative ion mode and 294 in the positive ion mode. A summary of some of the key components is provided in Table S5 and Figure S2, which are known for their diverse health benefits, including detoxification and hepatoprotection. To assess the efficacy of HSD in reducing cellular fat accumulation, we conducted in vitro experiments using a fatty acid-induced HepG2 cell model. Additionally, lipidomics analysis was performed to elucidate the hepatoprotective mechanisms of HSD, thereby providing empirical support for the network pharmacology predictions.

### 3.2. Improvement of Fatty Acid-Induced Lipid Accumulation in HepG2 Cells by HSD

To investigate the hepatoprotective effects of HSD, we employed the CCK-8 assay to evaluate the impact on HepG2 cell viability. The HSD concentrations tested were 0, 1.25, 2.5, 5, 7.5, 10, 15, and 20 μL/mL. HepG2 cells were co-cultured with HSD, followed by a CCK-8 assessment of cell viability.

**Figure 4:**
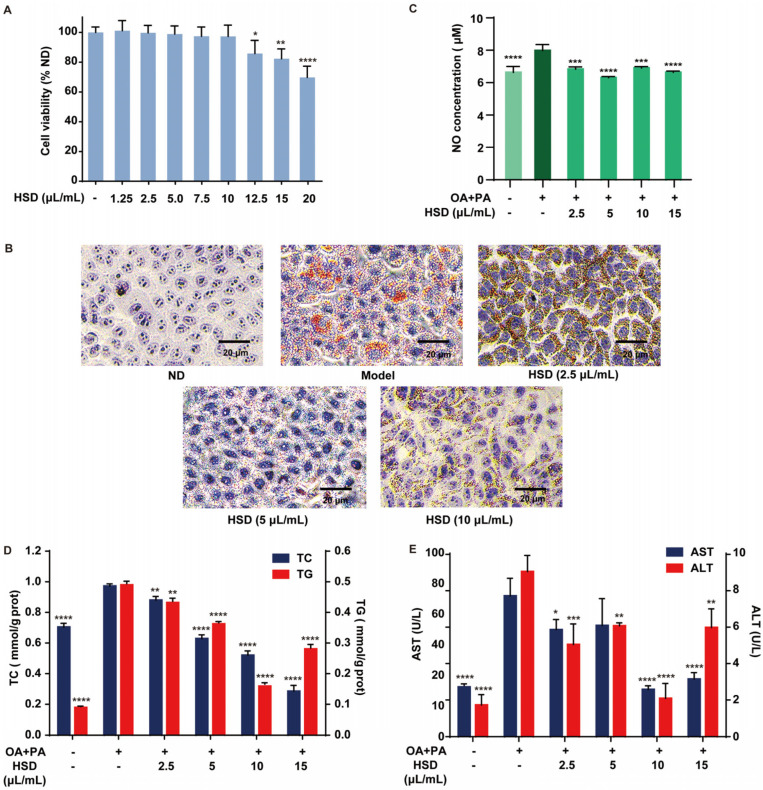
The protective effect of HepaSynergy Decoction (HSD) on fatty acid-induced cellular liver injury. (**A**) Inhibition rates of HSD on the proliferation of cells. Compared with the control group (ND), the difference was statistically significant; (**B**) oil red staining for observing lipid droplet accumulation in HepG2 cells; (**C**) the effect of HSD on NO expression in fatty acid-induced cells. Compared with the model group, it has significance; (**D**) the effect of HSD on TC and TG levels in cells; (**E**) the effect of HSD on ALT and AST levels due to cell damage. Compared with the model group, it has significance (*, *p* < 0.05; **, *p* < 0.01; ***, *p* < 0.005; ****, *p* < 0.001).

As shown in Figure 4A, the inhibition rate of HSD increases with the concentration. When the concentration reached 12.5 μL/mL HSD, the inhibition rate was 85.80 ± 8.87% (*p* < 0.05). At 20 μL/mL HSD, the inhibition rate significantly increased to 69.79 ± 7.53% (*p* < 0.001), while at concentrations below 10 μL/mL HSD, there was no significant effect on HepG2 cell viability. These data suggest that HSD exhibits maximal inhibition at high concentrations, with no significant impact on cell viability at concentrations below 10 μL/mL.

To investigate the impact of HSD on lipid content in HepG2 cells, we used the oil red O staining method to observe changes in lipid accumulation after different concentrations of HSD intervention (Figure 4B). Control cells presented with blue, with minimal lipid accumulation. Compared to the control group, the model group showed significant red lipid droplet formation. In contrast, HSD intervention reduced the number of lipid droplets. The effect was more pronounced in the 2.5 and 5 μL/mL HSD groups. This effect was more pronounced at 5 μL/mL, indicating a dose-dependent increase in efficacy. HSD inhibited the formation of free fatty acid-induced lipid droplets, though its effect diminished at 10 μL/mL.

### 3.3. Alleviation in Fatty Acid-Induced Liver Injury in HepG2 Cells by HSD

To investigate the impact of HSD on NO levels in HepG2 cells, we measured the NO content, a crucial messenger or effector molecule involved in various physiological and pathological responses. NO release in HepG2 cells serves as a marker for inflammatory responses, especially in liver diseases or infections [46]. We detected NO content in HepG2 cells (Figure 4C). Compared to the control group, the model group showed a significant increase in NO content. Following HSD treatment, NO levels were reduced, with no significant difference from the control group. These findings suggest that HSD alleviates inflammatory responses by reducing the NO content.

Liver cells absorb free fatty acids from the blood and synthesize TG de novo, which can lead to disease development when accumulated excessively in the liver cells [47]. To evaluate the impact of HSD on TG content in HepG2 cells, we measured TG levels (Figure 4D). The results show that TG levels are consistent among groups. Compared to the control group, TG content in the model group was significantly higher, approximately 5.49 times, confirming the successful establishment of a fatty liver cell model. TG levels significantly decreased in the HSD intervention groups at 5 and 10 μL/mL (*p* < 0.001), with the 10 μL/mL group exhibiting the greatest reduction (67%). However, at 15 μL/mL, TG levels increased, possibly due to increased free fatty acid entry into cells, reducing the lipid-lowering effect [48].

To assess the impact of HSD on TC content in HepG2 cells, we measured TC levels (Figure 4D). The results show that the TC content in model group cells is significantly higher than the control group, approximately 1.38 times, indicating abnormal lipid protein expression under free fatty acid intervention. Compared to the model group, TC levels in HSD intervention groups at 5, 10, and 15 μL/mL significantly decreased (*p* < 0.005), with a more pronounced effect at 10 μL/mL, reducing the TC content by 47%. This is consistent with the findings of Gao et al., suggesting HSD can reduce oxidative stress by decreasing the TC content, thereby lowering the risk of fatty liver development [49].

ALT and AST are typically present in hepatocytes; when hepatocytes are damaged, these enzymes are released into the bloodstream, resulting in increased plasma levels. Elevated AST and ALT activities are a key indicator of hepatocellular damage [50]. To evaluate the impact of HSD on hepatocyte damage, we measured ALT and AST enzyme activities in cell culture supernatants (Figure 4E). the results show significant increases of 184% and 426% (*p* < 0.001 and *p* < 0.01), respectively, in the model group compared to the control group, indicating hepatocellular damage. Compared to the model group, HSD intervention groups at 5, 10, and 15 μL/mL significantly reduced enzyme activity (*p* < 0.01 and *p* < 0.001), with more pronounced effects at 10 μL/mL, reducing activity by 66% and 76%. This suggests HSD has a beneficial effect on lipid metabolism, improving hepatocyte damage.

While higher concentrations generally exhibit more pronounced effects, our results underscore the need to balance efficacy with potential cytotoxicity. The CCK-8 assay indicated that HSD is well-tolerated by cells at concentrations up to 10 μL/mL; with no significant cytotoxicity observed, the cumulative effects of its bioactive components may enhance the overall efficacy as concentration increases. The concentration of 10 μL/mL appears to be the most effective for reducing lipid accumulation and inflammation without compromising cell viability.

### 3.4. Analysis of the Effect of HSD on Lipid Composition in Fatty Acid-Induced HepG2 Cells Based on Lipidomics

The liver is the central organ for lipid metabolism, and the disruption of the balance between lipid generation and consumption within the liver promotes the development of MAFLD. Initially, the liver experiences an accumulation of toxic lipids, such as TG and free fatty acids, which triggers a cascade of lipotoxic reactions. Over time, this chronic condition leads to alterations in hepatocytes, including the activation of hepatic stellate cells and the deposition of extracellular matrix in the liver tissue, resulting in pathological changes [51,52]. This injury–repair cycle generates a significant amount of free radicals in the liver, which is considered a critical step in the lipotoxicity of MAFLD [51]. This process further leads to lipid peroxidation in hepatocytes and aberrant signal transduction. Lipidomics is a valuable tool that aids in the identification of biomarkers and metabolic pathways associated with diseases [12,53,54,55]. Considering the significant impact of lipid changes on MAFLD, and the network pharmacology prediction results, we performed a lipidomics analysis on HepG2 cells under high-fat induction in the model and HSD intervention groups.

**Figure 5:**
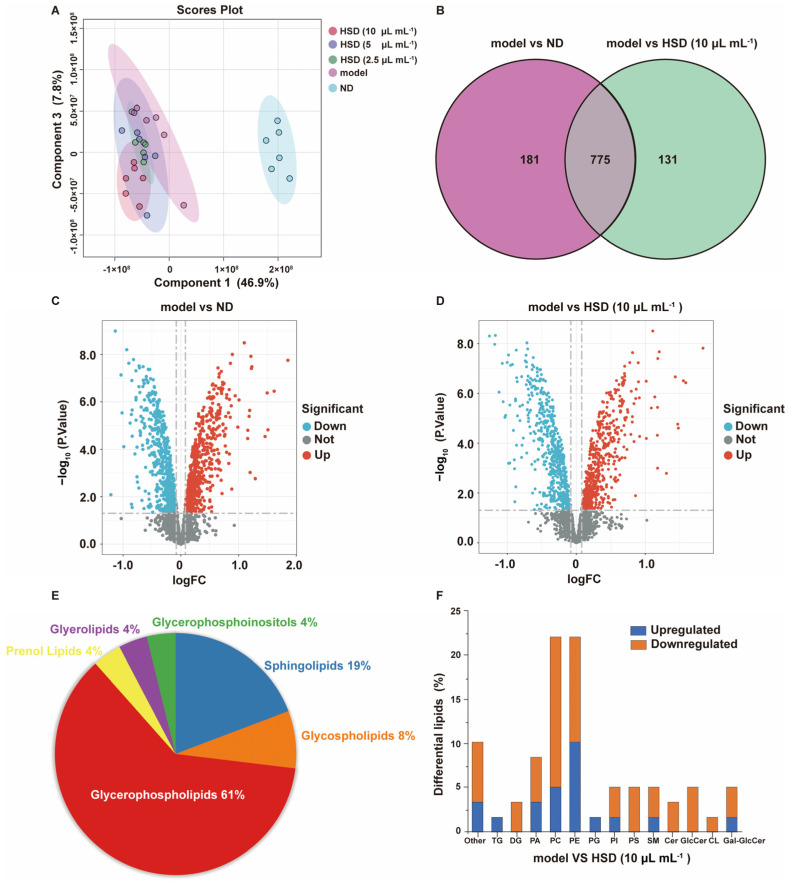
Lipidomics analysis results of fatty acid-induced cells treated with HepaSynergy Decoction (HSD). (**A**) PLS-DA of lipids in liver cells; (**B**) differential lipid metabolites in Venn diagram. A total of 956 metabolites differentially expressed between the control and model groups (in purple circle), and 906 metabolites between the HSD and model groups (in green circle), with 775 metabolites common to both comparisons; (**C**) volcano plot of lipid metabolites in the model and control groups. Metabolites with a significant fold change (*x*-axis) and statistical significance (*y*-axis) are highlighted; (**D**) volcano plot of lipid metabolites in the model and 10 μL/mL HSD groups; (**E**) distribution proportion of 59 lipid metabolites; (**F**) the proportion of changes in differential lipid metabolites. ND: control group; model: fatty acid-induced cells.

The stability and reproducibility of the samples were evaluated using Orthogonal Partial Least Squares Discriminant Analysis (OPLS-DA) and Partial Least Squares Discriminant Analysis (PLS-DA), as shown in Figure S3. The quality control samples showed good clustering within groups and distinct separation between groups, indicating the impact of high-fat induction and HSD intervention on cellular metabolic patterns. Figure 5A shows distinct clustering within groups, with HSD treatment reducing the effects of high-fat induction on lipid metabolism.

The results in Figure 5B show that there are 956 metabolites differentially expressed between the control and model groups, and 906 between the HSD and model groups, with 775 common metabolites. The volcano plot (Figure 5C,D) indicates that 18 metabolites were upregulated in the control group, 41 in the model group, and 41 downregulated in the 10 μL/mL HSD group, suggesting a significant impact of HSD on MAFLD, improving lipid metabolism. Notably, 59 metabolites are associated with lipid metabolism (Table S6). These differentially expressed metabolites may be involved in the protective mechanism of HSD against liver damage, warranting further investigation of their biological functions and pathways.

The enrichment analysis of the 59 lipid metabolites in Figure 5E and Table 1 reveals that the main pathways involved are sphingolipid and glycerophospholipid metabolism. The disruption of sphingolipid metabolism in hepatocyte membranes is a core feature of MAFLD progression to metabolic-associated steatohepatitis. Sphingolipid metabolism disruption not only promotes hepatic fat accumulation, but also exacerbates liver inflammation and fibrosis [56]. Glycerophospholipids, as crucial components of cell membranes, have their metabolic disturbances potentially impacting membrane integrity and function, thereby affecting lipid storage and metabolism. Furthermore, alterations in glycerophospholipid metabolism may be linked to insulin resistance and inflammatory responses, factors that collectively contribute to the onset and progression of MAFLD [54]. Compared to the model group (Figure 5F), intervention with 10 μL/mL HSD resulted in a significant reduction in TG and PG levels, while diglyceride (DG) levels, as a product of TG hydrolysis, increased. This indicates that HSD enhances lipolysis, inhibits lipid accumulation, improves lipid metabolism, and potentially reduces cardiovascular risk [53].

Ceramides (Cers) and glucosylceramides (GlcCers) are both crucial components of sphingolipid metabolism and have garnered significant attention in the study of MAFLD mechanisms. Ceramides can be converted into glucosylceramides through the action of glucosylceramide synthase, and this interconversion plays a significant role in lipid homeostasis and cellular function. The research indicates that ceramides can promote insulin resistance and the production of mitochondrial reactive oxygen species [57]. Notably, dihydroceramide, a ceramide precursor, has been linked to insulin resistance, and elevated levels of dihydroceramide have been observed in the livers of patients with Non-Alcoholic Steatohepatitis [58]. Modulating the dihydroceramide/ceramide ratio holds promise as a therapeutic target for MAFLD [59]. In our study, we observed elevated levels of Cer and GlcCer after intervention with 10 μL/mL HSD. This increase suggests a potential interplay between these two sphingolipid metabolites, where the elevation of ceramides may lead to enhanced conversion into glucosylceramides. This interconversion can contribute to the suppression of fat synthesis, promotion of lipolysis, and enhancement of lipid transport and utilization, thereby reducing fat accumulation. Additionally, the increase in Cer and GlcCer levels might modulate inflammatory signaling pathways, leading to a decrease in inflammatory factor release and alleviating liver inflammation [60]. We observed an increase in cardiolipin (CL) levels, and although there is a lack of direct research evidence confirming the impact of elevated CL levels on lipid metabolism and MAFLD, it may promote mitochondrial function and fatty acid oxidation, accelerating the breakdown and utilization of fatty acids in the liver, thus reducing hepatic fat accumulation [55]. This can help alleviate hepatic steatosis and mitigate liver damage. Concurrently, it may reduce the production of reactive oxygen species, thereby attenuating the liver’s inflammatory response [61].

The lipidomics analysis of fatty acid-induced HepG2 cells treated with HSD revealed significant alterations in key lipid components, including TG, sphingolipids, and glycerophospholipids. Consistent with the lipotoxicity theory, our findings indicate that DG and sphingolipids, particularly Cer and GlcCer, play a crucial role in promoting hepatic steatosis. The biological functions and pathways of these metabolites suggest that sphingolipid and glycerophospholipid metabolism pathways are involved in the pathogenesis of MAFLD, which is consistent with the results of our network pharmacology analysis. This underscores the complex interplay between glycerophospholipid metabolism and MAFLD, a connection that has not been extensively explored in the previous research. Our study demonstrates that HSD improves MAFLD by modulating these lipid metabolites, highlighting its potential as a therapeutic strategy for managing the disease.

## 4. Discussion

MAFLD has emerged as one of the most prevalent chronic liver diseases, significantly contributing to the development of cirrhosis and primary liver cancer. Despite its growing impact, the precise etiology of MAFLD remains elusive, posing significant challenges for the development of targeted clinical treatments. Many existing drugs exhibit limited efficacy and carry high side-effect profiles, underscoring the urgent need for alternative therapeutic approaches. Functional FMH approaches have demonstrated notable advantages in treating and preventing liver diseases, earning global recognition from scholars. These approaches leverage the synergistic benefits of natural food-based compounds, which are generally recognized as safe (GRAS) and have a long history of dietary use. This makes FMH-based interventions particularly suitable for long-term dietary strategies.

For instance, *H. dulcis* exhibits significant hepatoprotective properties. Compounds such as dihydromyricetin and naringenin, extracted from H. dulcis, enhance the activity of alcohol dehydrogenase and aldehyde dehydrogenase enzymes in the liver, facilitating alcohol metabolism and detoxification [33,34]. Naringenin, lutein, myricetin, and apigenin can activate and stimulate the production of inflammatory mediators like IL-1β, IL-6, TNF-α, COX-2, and inducible nitric oxide synthase, thereby inhibiting liver fibrosis progression [37]. *R. puerariae* is a promising food supplement for treating fatty liver diseases, exerting multi-pathway and multi-target effects by restoring intestinal barrier integrity and modulating gut microbiota [62,63,64]. *H. erinaceus* has shown significant therapeutic effects in mouse models of alcoholic liver injury [40], and its supplementation in chicken feed has improved liver lipid metabolism [41]. The research indicates that different preparation methods can influence FMH efficacy. For example, stir-fried *H. dulcis* seeds exhibit superior hepatoprotective effects compared to raw seeds, underscoring the impact of processing on nutritional benefits [32]. Preparing *H. dulcis* as juices and fermented vinegars also preserves its hepatoprotective properties [65]. Combining FMH with other herbs can produce synergistic effects. In cases of fatty liver, *C. oleifera* oil combined with other herbs is more effective in reducing lipid accumulation than *C. oleifera* oil alone [66]. *C. longa* is often combined with other FMH substances to produce various nutritional benefits. Clinically, the combination of *C. longa* and turtle shell mainly exhibits hepatoprotective effects, as seen in formulations like “Fugan Huaxian Decoction” and “Compound Biejia Ruangan Tablets”. When combined with Astragalus, it is primarily used to treat gynecological diseases, tumors, and digestive tract inflammation and cancer, including liver cancer [42]. Additionally, vinegar-processed *C. longa* has been found to be more effective in improving liver fibrosis than raw *C. longa* [43]. The judicious combination of FMH can enhance the beneficial effects of its natural bioactive compounds. FMH leverages the synergistic benefits of natural food-based compounds, which are generally recognized as safe and have a long history of dietary use. This makes FMH-based interventions particularly suitable for long-term dietary strategies. In this work, *R. puerariae*, *H. erinaceus*, *C. longa*, *C. oleifera* and *H. Dulcis* Semen not only provide therapeutic effects but also contribute to overall nutritional balance, supporting long-term health maintenance.

The sustained modulation of these pathways through a regular dietary intake of FMH-based compounds could potentially lead to long-term improvements in liver health and overall metabolic function. This aligns with the growing awareness of health and wellness, positioning FMH-based food supplements for rapid growth in the high-end health food and functional food markets. Future research should focus on exploring the interactions of various FMH active ingredients and their compatibility with other FMH. The complexity of multi-component interactions presents a challenge in fully elucidating the mechanisms of action of HSD. The synergistic and antagonistic interactions among the diverse compounds in HSD can significantly influence its overall therapeutic effect. For instance, certain compounds may enhance the activity of others, while some may counteract their effects. Understanding these interactions requires a systems biology approach that considers the dynamic interplay of multiple factors, including dose-dependent responses, temporal dynamics, and tissue-specific effects. Advances in the experimental and computational methods will clarify the active ingredients, target points, and mechanisms of action of FMH compound solutions for MAFLD. These natural plant or animal products hold the potential to treat diseases and enhance bodily resilience.

However, the development of FMH composite solutions based on the concept of FHM homology has not yet been standardized or industrialized. Overcoming the limitations of empirical methods and establishing systematic FMH composite solution preparation techniques is crucial. It is hoped that the findings of this work will provide a foundation for the development of new FMH food supplements and functional foods, with network pharmacology technology offering significant prospects for the prevention and treatment of diseases related to food with medicinal value.

## 5. Conclusions

This work leveraged network pharmacology to predict active ingredients and key targets for liver injury repair. Using SwissADME and Lipinski’s rule of five, with a focus on those from *R. puerariae*, *H. erinaceus*, *C. longa*, *C. oleifera*, and *H. Dulcis* Semen, the development of a FMH compound solution (HSD) with five medicinal and edible food products was proposed. The experimental validation in HepG2 cells demonstrated HSD hepatoprotective effects, including the inhibition of lipid accumulation, reduction in inflammatory markers, and improvement of hepatocyte damage. Our study primarily focused on the beneficial effects of HSD. It is also important to acknowledge that some compounds may interact with unintended targets, potentially leading to adverse effects. Future studies should aim to investigate these off-target interactions to ensure the safety and efficacy of HSD. Thus, by incorporating this additional investigation, particularly testing in animal models and clinical trials to provide a more comprehensive evaluation of HSD’s therapeutic potential, it is essential for the safe and effective development of HSD as a treatment for MAFLD. This work not only advances the understanding of the therapeutic potential of natural products in liver health, but also provides prospects for FMH as food-based MAFLD treatment strategies and a professional and reliable theoretical basis for the future development and research of function foods.

## Figures and Tables

**Table 1:** Metabolic pathway analysis with MetaboAnalyst from 57 lipid metabolites.

Pathway Name	Total	Hits	Expect	*p* Value	Holm *P*	FDR
Glycerophospholipids	40,000	5.0000	16	2.57 × 10^−6^	1.22 × 10^−3^	6.12 × 10^−4^
Sphingolipids	629	0.0787	5	1.56 × 10^−8^	7.41 × 10^−6^	7.41 × 10^−6^
Glycosphingolipids	13,400	1.6800	2	0.507	1.0	1.0
Glycerophosphoinositols	3190	0.3990	1	0.331	1.0	1.0
Prenol lipids	3830	0.4780	1	0.383	1.0	1.0
Glycerolipids	42,900	5.3600	1	0.998	1.0	1.0

Note: Total indicates the number of compounds in the pathway; hits reflect the degree of match between the uploaded data and the predefined metabolic pathways in the MetaboAnalyst database; expect value reflects the probability of observing specific metabolites in a random case; Holm *P* indicates the *p* value after the Holm–Bonferroni method adjustment; FDR indicates the false discovery rate after adjustment.

## Data Availability

The original contributions presented in the study are included in the article/Supplementary Material, further inquiries can be directed to the corresponding author.

## Supplementary Materials

The following supporting information can be downloaded at: https://www.mdpi.com/article/10.3390/foods14071257/s1, Table S1: Potential active ingredients for alleviating metabolic-associated fatty liver disease; Table S2: The top 20 pathways for KEGG pathway enrichment analysis; Table S3: The binding activity of 9 MAFLD-related genes and their corresponding 15 active ingredients; Table S4: Chemical structures of active ingredients with a binding energy < −7.0 kcal/mol in docking targets; Table S5: Metabolite identification of “HepaSynergy Decoction” by using the Agilent 6545 QTOF Mass Spectrometer; Table S6: 59 lipid metabolites identified by lipidomics analysis; Figure S1: A complex target pathway network of 52 main active ingredients. (Green diamonds represent targets, red hexagons represent active ingredients from *Rhizoma Curcumae longae*, purple hexagons represent active ingredients from *Hericium erinaceus*, blue hexagons represent active ingredients from *Hoveniae Dulcis* Semen, pink hexagons represent active ingredients from *Radix Puerariae*, yellow hexagons represent active ingredients from *Camellia oleifera*, and A1 is *Hericium erinaceus* and *Rhizoma Curcumae longae* posing overlapping active constants); Figure S2: Metabolite identification and classification of “HepaSynergy Decoction”. The inner circle has a positive spectrum, while the outer circle has a negative spectrum; Figure S3: (A) PLS-DA of lipids in high-fat-induced cells (model) and HepG2 cells (ND); (B) PLS-DA of lipids in high-fat-induced cells (model) treated with 10 μL mL^−1^ HepaSynergy Decoction (HSD).

## Abbreviations

The following abbreviations are used in this manuscript:

MAFLD	Metabolic-associated fatty liver disease
FMH	Food and Medicine Homology
HSD	HepaSynergy Decoction
PPI	Protein–Protein Interaction
TG	Triglyceride
TC	Total Cholesterol
ALT	Alanine Aminotransferase
AST	Aspartate Aminotransferase
OA	Oleic Acid
PA	Palmitic Acid
AKT1	Protein Kinase B
PIK3R1	Phosphoinositide-3-Kinase Regulatory Subunit 1
MAPK8	Mitogen-Activated Protein Kinase 8
MAPK14	Mitogen-Activated Protein Kinase 14
NFKB1	Nuclear Factor Kappa B Subunit 1
IL-1β	Interleukin 1 Beta
IL-6	Interleukin 6
TNF-α	Tumor Necrosis Factor Alpha
COX-2	Cyclooxygenase-2
iNOS	Inducible Nitric Oxide Synthase
OPLS-DA	Orthogonal Partial Least Squares Discriminant Analysis
PLS-DA	Partial Least Squares Discriminant Analysis
DG	Diglyceride
Cer	Ceramide
GlcCer	Glucosylceramide
CL	Cardiolipin
GRAS	Generally recognized as safe
