# Supplementary material for: Exploring the Nutraceutical Potential of a Food–Medicine Compound for Metabolic-Associated Fatty Liver Disease via Lipidomics and Network Pharmacology
Source: Foods. 2025 Apr 3;14(7):1257. doi: 10.3390/foods14071257 (PMC11988326; doi:10.3390/foods14071257)
Supplement: Supplementary file 1 [file foods-14-01257-s001.zip › foods-3521986-supplementary.pdf]

*Supporting Information*

# Exploring the Nutraceutical Potential of a Food-Medicine Compound for Metabolic-Associated Fatty Liver Disease via Lipidomics and Network Pharmacology

Yuru Deng [1], Jie Cui [1, 2*], Yuxuan Jiang [3], Jian Zhang [1], Jinchi Jiang [1], Quanbin Zhang [2], and Yonghong Hu [2, 3]

1 College of Food Science and Light Industry, Nanjing Tech University, Nanjing 211816, China.; dengyuru-@njtech.edu.cn (Y.D.); jiecui_njtech@163.com (J.C.); four-leaf-clover@njtech.edu.cn (J.Z.); jiangjinchi@126.com (J.J.);

2 Institute of Oceanology, Chinese Academy of Sciences, Qingdao 266071, China; qbzhang@qdio.ac.cn (Q.Z.)

3 College of Biotechnology and Pharmaceutical Engineering, Nanjing Tech University, Nanjing 211816, China. jyxxxx@njtech.edu.cn (Y.J.); yonghonghuyg@163.com (Y.H.).

* Correspondence: jiecui_njtech@163.com (J.C.).

**Table S1.** Potential active ingredients for alleviating Metabolic-associated fatty liver disease.

| Food and medicine homology | Label | Mol ID | Molecule name |
|---|---|---|---|
| Radix Puerariae (HERB001874) | GG1 | MOL000392 | Formononetin |
| | GG2 | MOL000481 | Genistein |
| | GG3 | MOL012976 | Coumestrol |
| | GG4 | MOL000390 | Daidzein |
| | GG5 | MOL002959 | 3'-Methoxydaidzein |
| | GG6 | MOL001837 | Methyl 4-hydroxycinnamate |
| | GG7 | MOL004631 | 7,8,4'-Trihydroxyisoflavone |
| | GG8 | MOL002964 | (S)-4-Methoxydalbergione |
| | GG9 | MOL000391 | 3-(4-methoxyphenyl)-7-[(2S,4S,5R)-3,4,5-trihydroxy-6-(hydroxymethyl)oxan-2-yl]oxychromen-4-one |
| Hericium erinaceus (HERB002345) | HTG1 | MOL000254 | Eugenol |
| | HTG2 | MOL000703 | 2-Heptanone |
| | HTG3 | MOL004687 | 2-Octanone |
| | HTG4 | MOL004664 | Heptanoic Acid |

| | | | |
|---|---|---|---|
| | HTG5 | MOL000705 | Heptanal |
| | HTG6 | MOL003050 | Nonanoic acid |
| | HTG7 | MOL000924 | 2-Undecanone |
| | HTG8 | MOL008165 | 2-Decanone |
| | HTG9 | MOL000918 | 2-Nonanone |
| | HTG10 | MOL000116 | Nonanal |
| | HTG11 | MOL000206 | Isoeugenol |
| | HTG12 | MOL004480 | n-(4-Chlorobenzoyl)-melatonin |
| Rhizoma Curcumae longae (HERB002840) | JH1 | MOL000303 | Octanoic acid |
| | JH2 | MOL000305 | Lauric Acid |
| | JH3 | MOL005101 | Nitidine |
| | JH4 | MOL003915 | Phenylacetonitrile |
| | JH5 | MOL005102 | Oxynitidine |
| | JH6 | MOL000961 | Procurcumenol |
| | JH7 | MOL002485 | 4-(1,5-Dimethylhex-4-enyl)cyclohex-2-enone |
| | JH8 | MOL000090 | Curcumin |
| | JH9 | MOL000166 | (+)-Epi-alpha-bisabolol |
| | JH10 | MOL000940 | Bisdemethoxycurcumin |
| | JH11 | MOL002468 | Hexahydrocurcumin |
| | JH12 | MOL000946 | Demethoxycurcumin |
| | JH13 | MOL000244 | Borneol |
| | JH14 | MOL000892 | (1Z,6Z)-1,7-bis(4-hydroxy-3-methoxyphenyl)hepta-1,6-diene-3,5-dione |
| | JH15 | MOL004252 | 1,7-Bis(4-hydroxyphenyl)-1-heptene-3,5-dione |
| | JH16 | MOL000951 | (1E)-1,7-bis(4-hydroxy-3-methoxyphenyl)hept-1-ene-3,5-dione |
| | JH17 | MOL004312 | 1,7-Bis(4-hydroxy-3-methoxyphenyl)-1,4,6-heptatrien-3-one |
| | JH18 | MOL000941 | Zingiberenol |
| Camellia oleifera | SCY1 | MOL000255 | Tetradecanoic acid |

| | | | |
|---|---|---|---|
| (HERB006672) | SCY2 | MOL001558 | sesamin |
| | SCY3 | MOL002295 | cinnamic acids |
| | SCY4 | MOL010246 | flavonols |
| Hoveniae Dulcis Semen (HERB007002) | ZJZ1 | MOL000513 | Gallic Acid |
| | ZJZ2 | MOL000472 | Emodin |
| | ZJZ3 | MOL000114 | Vanillic Acid |
| | ZJZ4 | MOL007233 | Methyl nonanoate |
| | ZJZ5 | MOL006844 | 9H-Pyrido[3,4-B]indole |
| | ZJZ6 | MOL004328 | (2R)-5,7-dihydroxy-2-(4-hydroxyphenyl)-2,3-dihydro-4H-chromen-4-one |
| | ZJZ7 | MOL000008 | Apigenin |
| | ZJZ8 | MOL013375 | Hovenine A |
| Overlapping active constituent of Hericium erinaceus and Rhizoma Curcumae longae | A1 | MOL000121 | Decanal |

**Table S2.** The top 20 pathways for KEGG pathway enrichment analysis.

| GO | Description | LogP |
|---|---|---|
| hsa05200 | Pathways in cancer | -34.3111 |
| hsa05207 | Chemical carcinogenesis - receptor activation | -27.5844 |
| hsa05417 | Lipid and atherosclerosis | -27.4773 |
| hsa05161 | Hepatitis B | -25.1248 |
| hsa05167 | Kaposi sarcoma-associated herpesvirus infection | -23.9096 |
| hsa04933 | AGE-RAGE signaling pathway in diabetic complications | -23.6579 |
| hsa04917 | Prolactin signaling pathway | -23.3374 |
| hsa05163 | Human cytomegalovirus infection | -22.9179 |
| hsa05205 | Proteoglycans in cancer | -21.468 |
| hsa04066 | HIF-1 signaling pathway | -20.8796 |
| hsa04926 | Relaxin signaling pathway | -19.9639 |
| hsa05235 | PD-L1 expression and PD-1 checkpoint pathway in cancer | -19.6897 |
| hsa05215 | Prostate cancer | -19.257 |
| hsa04932 | Non-alcoholic fatty liver disease | -18.9752 |
| hsa05208 | Chemical carcinogenesis - reactive oxygen species | -18.9655 |
| hsa04625 | C-type lectin receptor signaling pathway | -18.9084 |
| hsa05160 | Hepatitis C | -18.9065 |
| hsa05145 | Toxoplasmosis | -18.5392 |
| hsa05152 | Tuberculosis | -18.1763 |
| hsa05212 | Pancreatic cancer | -18.1692 |

**Table S3** The binding activity of 9 MAFLD-related genes and their corresponding 15 active ingredients.

| Targets | Active ingredients | Affinity (kcal/mol) | Targets | Active ingredients | Affinity (kcal mol$^{-1}$) |
|---|---|---|---|---|---|
| AKT1 | GG3 | -10.315 | STAT3 | JH17 | -5.537 |

| | | | | | |
|---|---|---|---|---|---|
| AKT1 | JH8 | -10.02 | STAT3 | HTG11 | -5.019 |
| AKT1 | ZJZ7 | -9.413 | STAT3 | JH14 | -4.832 |
| AKT1 | JH14 | -9.23 | STAT3 | JH8 | -4.568 |
| AKT1 | JH11 | -8.754 | STAT3 | JH16 | -4.221 |
| PIK3R1 | ZJZ7 | -8.829 | STAT3 | ZJZ4 | -2.842 |
| PIK3R1 | JH11 | -8.119 | MAPK8 | JH5 | -8.292 |
| PIK3R1 | ZJZ4 | -4.905 | MAPK8 | GG8 | -6.17 |
| PIK3CA | JH3 | -4.049 | MAPK8 | ZJZ4 | -3.55 |
| PIK3CA | JH5 | -3.753 | MAPK14 | JH3 | -8.804 |
| PIK3CA | ZJZ6 | -3.274 | MAPK14 | GG9 | -6.967 |
| MAPK3 | JH11 | -5.781 | MAPK14 | GG8 | -5.932 |
| RELA | HTG11 | -3.89 | MAPK14 | HTG7 | -3.771 |
| NFKB1 | GG3 | -7.164 | | | |

**Table S4** Chemical Structures of Active Ingredients with Binding Energy < -7.0 kcal/mol in Docking Targets.

| Active ingredients | Mol ID | PubChem CID | Molecule name | Chemical Structure |
|---|---|---|---|---|

| | | | | |
|---|---|---|---|---|
| GG3 | MOL012976 | 5281707 | Coumestrol | 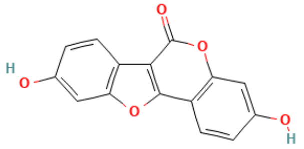 |
| ZJZ7 | MOL000008 | 5280443 | Apigenin | 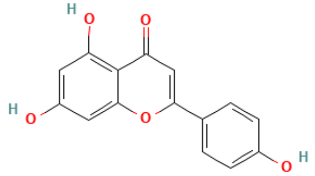 |
| JH3 | MOL005101 | 4501 | Nitidine | 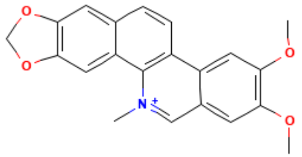 |
| JH5 | MOL005102 | 97597 | Oxynitidine | 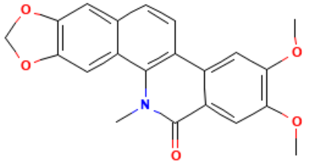 |

| | | | | |
|---|---|---|---|---|
| JH8 | MOL000090 | 5281767 | Curcumin | 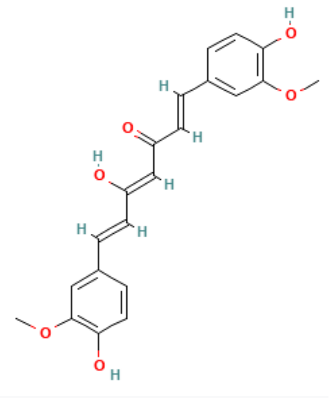 |
| JH11 | MOL002468 | 5318039 | Hexahydrocurcumin | 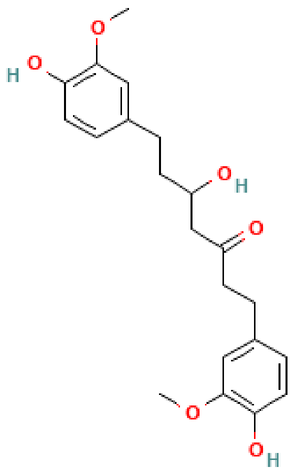 |

| | | | | |
|---|---|---|---|---|
| JH14 | MOL000892 | 6604598 | (1Z,6Z)-1,7-bis(4-hydroxy-3-methoxyphenyl)hepta-1,6-diene-3,5-dione | 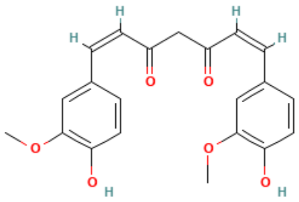 |

**Table S5** Metabolite Identification of "HepaSynergy Decoction" by using Agilent 6545 QTOF Mass Spectrometer.

| **Synonym** | **RT (min)** | **Formula** | **Adduct** | **Class** | **type** | **Synonym** | **RT (min)** | **Formula** | **Adduct** | **Class** | **type** |
|---|---|---|---|---|---|---|---|---|---|---|---|
| prol | 9.9 | C5H9NO2 | [M + H]+ | Alpha amino acids and derivatives | pos | phenylalaninate | 2.8 | C9H11NO2 | [M - H]- | Alpha amino acids and derivatives | neg |
| histidinediium | 0.8 | C6H9N3O2 | [M + H]+ | Alpha amino acids and derivatives | pos | Cumoestrol | 24.3 | C15H8O5 | [M - H]- | Coumestans | neg |
| meritonin | 1.8 | C5H11NO2S | [M + H]+ | Alpha amino acids and derivatives | pos | curcumin | 25.2 | C21H20O6 | [M - H]- | Curcuminoids | neg |
| Hexadecanamide | 32.7 | C16H33NO | [M + H]+ | Carboximidic acids | pos | Demethoxycurcumin | 24.9 | C20H18O5 | [M - H]- | Curcuminoids | neg |
| 1-Stearoyl-2-myristoyl-sn- | 34.2 | C35H68O5 | [M + Na]+ | Diacylglycerols | pos | Valylleucine | 5.2 | C11H22N2O3 | [M - H]- | Dipeptides | neg |

| | | | | | | | | | | | |
|---|---|---|---|---|---|---|---|---|---|---|---|
| glycerol | | | | | | | | | | | |
| 1-eicosenoyl-2-palmitoleoyl-sn-glycerol | 33 | C39H72O5 | [M + H]+ | Diacylglycerols | pos | Alanylphenylalanine | 4.8 | C12H16N2O3 | [M - H]- | Dipeptides | neg |
| Methionylmethionine | 4.1 | C10H20N2O3S2 | [M + H]+ | Dipeptides | pos | serylleucine | 3 | C9H18N2O4 | [M - H]- | Dipeptides | neg |
| isoleucylphenylalanine | 10 | C15H22N2O3 | [M + H]+ | Dipeptides | pos | leucylthreonine | 1.5 | C10H20N2O4 | [M - H]- | Dipeptides | neg |
| Serylmethionine | 1.6 | C8H16N2O4S | [M + H]+ | Dipeptides | pos | leucylglycine | 2.1 | C8H16N2O3 | [M - H]- | Dipeptides | neg |
| threonylphenylalanine | 4.9 | C13H18N2O4 | [M + H]+ | Dipeptides | pos | Leucylleucine | 8.4 | C12H24N2O3 | [M - H]- | Dipeptides | neg |
| threonylmethionine | 1.8 | C9H18N2O4S | [M + H]+ | Dipeptides | pos | Valylvaline | 2.3 | C10H20N2O3 | [M - H]- | Dipeptides | neg |
| Alanyltyrosine | 2.3 | C12H16N2O4 | [M + H]+ | Dipeptides | pos | leucylphenylalanine | 11.2 | C15H22N2O3 | [M - H]- | Dipeptides | neg |
| Erucylamide | 32.2 | C22H43NO | [M + H]+ | Fatty amides | pos | Glycylphenylalanine | 4.7 | C11H14N2O3 | [M - H]- | Dipeptides | neg |
| Oleamide | 32.8 | C18H35NO | [M + H]+ | Fatty amides | pos | Asparaginylleucine | 3.2 | C10H19N3O4 | [M - H]- | Dipeptides | neg |
| rutina | 13.3 | C27H30O16 | [M + Na]+ | Flavonoid O-glycosides | pos | pyroglutamylvalinate | 4.6 | C10H16N2O4 | [M - H]- | Dipeptides | neg |
| Ononin | 18.2 | C22H22O9 | [M + H]+ | Isoflavonoid O-glycosides | pos | threonylleucine | 3.4 | C10H20N2O4 | [M - H]- | Dipeptides | neg |
| daidsin | 11 | C21H20O9 | [M + H]+ | Isoflavonoid | pos | Ilixathin | 13.3 | C27H30O16 | [M - H]- | Flavonoid | neg |

| | | | | | | | | | | | |
|---|---|---|---|---|---|---|---|---|---|---|---|
| | | | | O-glycosides | | | | | | O-glycosides | |
| 1-g-linolenoyl-2-linoleoyl-sn-glycerol | 34.9 | C39H66O5 | [M + H]+ | Lineolic acids and derivatives | pos | daidzein | 19.4 | C15H10O4 | [M - H]- | Isoflavones | neg |
| Palmitoyllysolectithin | 30.3 | C24H50NO7P | [M + H]+ | Lysophosphatidylcholines | pos | genistein | 24 | C15H10O5 | [M - H]- | Isoflavones | neg |
| Phthalsaeureanhydrid | 31.6 | C8H4O3 | [M + H]+ | Phthalic anhydrides | pos | 3-Genistein-8-C-glucoside | 12 | C21H20O10 | [M - H]- | Isoflavonoid C-glycosides | neg |
| Octadecanamide | 34 | C18H37NO | [M + H]+ | Carboxylic acids and derivatives | pos | carboxyribitol | 0.9 | C6H12O7 | [M - H]- | Medium-chain hydroxy acids and derivatives | neg |
| Valylglutamine | 1.1 | C10H19N3O4 | [M + H]+ | Dipeptides | pos | Calycosin | 20.2 | C16H12O5 | [M - H]- | O-methylated isoflavonoids | neg |
| alanylmethionine | 1.8 | C8H16N2O3S | [M + H]+ | Dipeptides | pos | formononetin | 25.9 | C16H12O4 | [M - H]- | O-methylated isoflavonoids | neg |
| Tricosanamide | 32.4 | C23H47NO | [M + H]+ | Fatty amides | pos | alanylleucine | 3.3 | C9H18N2O3 | [M - H]- | Peptides | neg |
| Monooleoylglycero | 32.9 | C21H40O4 | [M + H]+ | Monoacylglyc | pos | Corylin | 28.7 | C20H16O4 | [M - H]- | Pyranoisofl | neg |

| | | | | | | | | | | | |
|---|---|---|---|---|---|---|---|---|---|---|---|
| l | | | | erols | | | | | | avonoids | |
| Monopalmitin | 32.7 | C19H38O4 | [M + H]+ | Monoacylglycerols | pos | Citrate | 1.4 | C6H8O7 | [M - H]- | Tricarboxylic acids and derivatives | neg |
| Cyclo-L-leu-L-pro | 10.4 | C11H18N2O2 | [M + H]+ | Alpha amino acids and derivatives | pos | pantothenate | 3.3 | C9H17NO5 | [M - H]- | Beta amino acids and derivatives | neg |
| cFL | 18.7 | C15H20N2O2 | [M + H]+ | Alpha amino acids and derivatives | pos | leucylserine | 1.4 | C9H18N2O4 | [M - H]- | Dipeptides | neg |
| 1-vaccenoyl-2-palmitoyl-sn-glycerol | 34.5 | C37H70O5 | [M + Na]+ | Diacylglycerols | pos | le | 1.9 | C11H20N2O5 | [M - H]- | Dipeptides | neg |
| Valylalanine | 1.4 | C8H16N2O3 | [M + H]+ | Dipeptides | pos | leucylglutamine | 1.5 | C11H21N3O4 | [M - H]- | Dipeptides | neg |
| wistin | 19.9 | C23H24O10 | [M + H]+ | Isoflavonoid O-glycosides | pos | malate | 1.1 | C4H6O5 | [M - H]- | Beta hydroxy acids and derivatives | neg |
| methionylleucine | 6.8 | C11H22N2O3S | [M + H]+ | Dipeptides | pos | Bisdemethoxycurcumin | 24.6 | C19H16O4 | [M - H]- | Curcuminoids | neg |
| (2S)-2-[(2R,3R)-2-[(2S)-2-{[(2S)-1-[(2R,3R)-2-amino-3-methylpentanoyl]p | 19.9 | C32H51N5O7 | [M + H]+ | Peptides | pos | isoleucylleucine | 7.6 | C12H24N2O3 | [M - H]- | Dipeptides | neg |

| | | | | | | | | | | | |
|---|---|---|---|---|---|---|---|---|---|---|---|
| yrrolidin-2-yl]formamido}-3-(4-hydroxyphenyl)propanamido]-3-methylpentanamido]-4-methylpentanoic acid | | | | | | | | | | | |
| Lauryldiethanolamine | 26.6 | C16H35NO2 | [M + H]+ | Quaternary ammonium salts | pos | Vitexin 2''-O-xyloside | 12.2 | C26H28O14 | [M - H]- | Flavonoid C-glycosides | neg |
| Dl-ar-Turmerone | 31.2 | C15H20O | [M + H]+ | Sesquiterpenoids | pos | Neobavaisoflavone | 27.6 | C20H18O4 | [M - H]- | Isoflavones | neg |
| 1-(9Z-pentadecenoyl)-2-nonadecanoyl-sn-glycerol | 33.7 | C37H70O5 | [M + Na]+ | Diacylglycerols | pos | Puerarin | 12.8 | C21H20O9 | [M - H]- | Isoflavonoid C-glycosides | neg |
| Sissotrine | 22.7 | C22H22O10 | [M + H]+ | Isoflavonoid O-glycosides | pos | dimorphecolate | 30.7 | C18H32O3 | [M - H]- | Lineolic acids and derivatives | neg |
| Tyr-Pro-Gln | 3.8 | C19H26N4O6 | [M + H]+ | Peptides | pos | Ile-Ala-Leu | 10.9 | C15H29N3O4 | [M - H]- | Peptides | neg |
| ZIUSSTSXXLLKKK-KOBPDPAPSA-N | 32 | C21H20O6 | [M + H]+ | Curcuminoids | pos | Leu-Leu-Gln | 5.2 | C17H32N4O5 | [M - H]- | Peptides | neg |
| Histidylleucine | 1.4 | C12H20N4 | [M + H]+ | Dipeptides | pos | Spinosin | 6.3 | C28H32O15 | [M - H]- | Flavonoid | neg |

| | | | | | | | | | | | |
|---|---|---|---|---|---|---|---|---|---|---|---|
| | | O3 | | | | | | | | C-glycosides | |
| 2-linoleoyl-sn-glycero-3-phosphocholine | 29.7 | C26H50NO7P | [M + H]+ | Lysophosphatidylcholines | pos | Orita-13 | 16.2 | C15H10O5 | [M - H]- | Isoflavones | neg |
| Phe-Met-Pro | 10.2 | C19H27N3O4S | [M + H]+ | Peptides | pos | Asn-Gln-Leu | 3.6 | C15H27N5O6 | [M - H]- | Peptides | neg |
| Icosanamide | 31.9 | C20H41NO | [M + H]+ | Fatty amides | pos | 8-[3,5-dihydroxy-6-(hydroxymethyl)-4-(3,4,5-trihydroxyoxan-2-yl)oxyoxan-2-yl]-5,7-dihydroxy-3-(4-hydroxyphenyl)chromen-4-one | 12.5 | C26H28O14 | [M - H]- | Isoflavonoid C-glycosides | neg |
| Neovitexin | 7.1 | C21H20O10 | [M + H]+ | Flavonoid C-glycosides | pos | Ser-Val-Leu | 7.4 | C14H27N3O5 | [M - H]- | Peptides | neg |
| Lanceolarin | 21.2 | C27H30O14 | [M + H]+ | Isoflavonoid O-glycosides | pos | Isoprunetin | 24.4 | C16H12O5 | [M - H]- | Isoflavones | neg |
| isoleucylarginine | 1.6 | C12H25N5O3 | [M + H]+ | Peptides | pos | 3invertedexclamationmark-Methoxypuerarin | 13.3 | C22H22O10 | [M - H]- | Isoflavonoid C-glycosides | neg |

| | | | | | | | | | | | |
|---|---|---|---|---|---|---|---|---|---|---|---|
| Leucylmethionine | 5.5 | C11H22N2O3S | [M + H]+ | Dipeptides | pos | Ser-Ala-Leu | 4.2 | C12H23N3O5 | [M - H]- | Peptides | neg |
| Leucylalanine | 2 | C9H18N2O3 | [M + H]+ | Dipeptides | pos | Val-Leu-Gln | 3.2 | C16H30N4O5 | [M - H]- | Peptides | neg |
| Daidzein-4,7-diglucoside | 6.9 | C27H30O14 | [M + H]+ | Isoflavonoid O-glycosides | pos | isoleucylthreonine | 1.6 | C10H20N2O4 | [M - H]- | Dipeptides | neg |
| threonyltyrosine | 2.4 | C13H18N2O5 | [M + H]+ | Peptides | pos | Gln-Val-Leu | 8.3 | C16H30N4O5 | [M - H]- | Peptides | neg |
| LEU-ILE-THR | 6.7 | C16H31N3O5 | [M + H]+ | Peptides | pos | Leuzin | 1.7 | C6H13NO2 | [M - H]- | Alpha amino acids and derivatives | neg |
| Ser-Leu-Gln | 3.7 | C14H26N4O6 | [M + H]+ | Peptides | pos | Isoswertisin 2''-O-beta-arabinoside | 10.2 | C27H30O14 | [M - H]- | Flavonoid C-glycosides | neg |
| Thr-Ala-Leu | 5 | C13H25N3O5 | [M + H]+ | Peptides | pos | Violarvensin | 5.8 | C27H30O14 | [M - H]- | Flavonoid C-glycosides | neg |
| Allobetaxanthin | 34.6 | C40H54O | [M + H]+ | Triterpenoids | pos | Glu-Leu-Asn | 4.3 | C15H26N4O7 | [M - H]- | Peptides | neg |
| palmityldiethanolamine | 29.4 | C20H43NO2 | [M + H]+ | Alkanolamines | pos | Isoferulate | 16.3 | C10H10O4 | [M - H]- | Hydroxycinnamic acids | neg |
| Ala-Leu-Gln | 2.1 | C14H26N4O5 | [M + H]+ | Peptides | pos | 8-Prenylwighteone | 29.9 | C25H26O5 | [M - H]- | Isoflavanones | neg |
| Tyr-Pro-Ile-Pro-Phe | 15.2 | C34H45N5 | [M + H]+ | Peptides | pos | Leu-Leu-Pro | 8.9 | C17H31N3O4 | [M - H]- | Peptides | neg |

| | | | | | | | | | | | |
|---|---|---|---|---|---|---|---|---|---|---|---|
| | | O7 | | | | | | | | | |
| berberine | 35.4 | C20H17NO 4 | [M + H]+ | Protoberberine alkaloids and derivatives | pos | Gln-Leu-Phe | 12.7 | C20H30N4O5 | [M - H]- | Peptides | neg |
| beta-Amyrenonol | 35.1 | C30H48O2 | [M + H]+ | Triterpenoids | pos | p-Vinylphenol | 27.8 | C8H8O | [M - H]- | Styrenes | neg |
| (1E,4Z,6E)-5-hydroxy-1,7-bis(4-hydroxyphenyl)hepta-1,4,6-trien-3-one | 24.5 | C19H16O4 | [M + H]+ | Curcuminoids | pos | OCNYGKNIVPVPPX-UHFFFAOYSA-N | 16.4 | C10H10O4 | [M - H]- | Coumaric acids and derivatives | neg |
| 1,7-bis(4-hydroxy-3-methoxyphenyl)hept-1-ene-3,5-dione | 28.1 | C21H22O6 | [M + H]+ | Curcuminoids | pos | Ile-Ala-Ala | 3 | C12H23N3O4 | [M - H]- | Peptides | neg |
| Phe-Pro-Gln | 6.7 | C19H26N4 O5 | [M + H]+ | Peptides | pos | Ile-Ile-Gly | 4.6 | C14H27N3O4 | [M - H]- | Peptides | neg |
| Kudzusaponin SA4 | 21.2 | C47H74O2 0 | [M + H]+ | Triterpene saponins | pos | Asn-Leu-Pro | 3.8 | C15H26N4O5 | [M - H]- | Peptides | neg |
| Kyanmethin | 35.5 | C6H9N3 | [M + H]+ | Aminopyrimidines and derivatives | pos | Gln-Val-Pro | 3.8 | C15H26N4O5 | [M - H]- | Peptides | neg |
| Elaol | 31.6 | C16H22O4 | [M + H]+ | Benzoic acid esters | pos | Ile-Ala-Ser | 1.6 | C12H23N3O5 | [M - H]- | Peptides | neg |
| Isoleucylproline | 4.2 | C11H20N2 O3 | [M + H]+ | Dipeptides | pos | Chrysin 6-C-alpha-L- | 9.8 | C26H28O13 | [M - H]- | Flavonoid C- | neg |

| | | | | | | | | | | | |
|---|---|---|---|---|---|---|---|---|---|---|---|
| | | | | | | arabinopyranoside-8-C-glucoside | | | | glycosides | |
| Diethylhexylphthalate | 34 | C24H38O4 | [M + H]+ | Benzoic acid esters | pos | Betanaphthol | 27.8 | C10H8O | [M - H]- | Naphthols and derivatives | neg |
| leucinate | 1.7 | C6H13NO2 | [M + H]+ | Alpha amino acids and derivatives | pos | 8-[(2R,3R,4S,5S,6R)-4,5-dihydroxy-6-(hydroxymethyl)-3-[(2R,3S,4S,5S,6R)-3,4,5-trihydroxy-6-methyloxan-2-yl]oxyoxan-2-yl]-5,7-dihydroxy-2-(4-hydroxyphenyl)-6-[(2S,3R,4S,5R)-3,4,5-trihydroxyoxan-2-yl]chromen-4- | 6.7 | C32H38O18 | [M - H]- | Flavonoid C-glycosides | neg |

| | | | | | | | | | | | |
|---|---|---|---|---|---|---|---|---|---|---|---|
| | | | | | | one | | | | | |
| Molludistin 2''-O-glucoside | 5.7 | C27H30O14 | [M + H]+ | Flavonoid C-glycosides | pos | 4'-Methoxypuerarin | 14.8 | C22H22O9 | [M - H]- | Isoflavonoid C-glycosides | neg |
| n-methylerucamide | 34.4 | C23H45NO | [M + H]+ | N-acyl amines | pos | IristectorinB | 11.8 | C23H24O12 | [M - H]- | Isoflavonoid O-glycosides | neg |
| LEU-ILE-ILE | 15.6 | C18H35N3O4 | [M + Na]+ | Peptides | pos | Ile-Val-Pro | 7.4 | C16H29N3O4 | [M - H]- | Peptides | neg |
| (+)-Puerol B 2''-O-glucoside | 17.9 | C24H26O10 | [M + H]+ | Phenolic glycosides | pos | Pyroglutamate | 1.4 | C5H7NO3 | [M - H]- | Alpha amino acids and derivatives | neg |
| 2-[(2-amino-3-phosphonooxypropanoyl)amino]-4-methylpentanoic acid | 10.7 | C9H19N2O7P | [M + H]+ | Dipeptides | pos | (2S)-2-[[(2S)-2-amino-5-oxo-5-[[(2S)-5-oxopyrrolidine-2-carbonyl]amino]pentanoyl]amino]-3-phenylpropanoic acid | 9.6 | C19H24N4O6 | [M - H]- | Dipeptides | neg |
| (2R)-3-(4-hydroxy-2-methoxyphenyl)- | 24.4 | C18H16O5 | [M + H]+ | Methoxyphenols | pos | Galangin | 20.2 | C15H10O5 | [M - H]- | Flavonols | neg |

| | | | | | | | | | | | |
|---|---|---|---|---|---|---|---|---|---|---|---|
| 2-[(4-hydroxyphenyl)methyl]-2H-furan-5-one | | | | | | | | | | | |
| SER-LEU-ILE | 5.3 | C15H29N3O5 | [M + H]+ | Peptides | pos | 5-hydroxy-3-(4-hydroxy-3-methoxyphenyl)-6-methoxy-7-[3,4,5-trihydroxy-6-[[3,4,5-trihydroxy-6-(hydroxymethyl)oxan-2-yl]oxymethyl]oxan-2-yl]oxychromen-4-one | 7.5 | C29H34O17 | [M - H]- | Isoflavonoid O-glycosides | neg |
| 2,3-bis[[(6Z,9Z)-dodeca-6,9-dienoyl]oxy]propyl dodecanoate | 34.7 | C39H66O6 | [M + H]+ | Triacylglycerols | pos | 3-(4-hydroxy-3-methoxyphenyl)-5-methoxy-7-[(2S,3R,4S,5S,6R)-3,4,5-trihydroxy-6-(hydroxymethyl | 18.2 | C23H24O11 | [M - H]- | Isoflavonoid O-glycosides | neg |

| | | | | | | | | | | | |
|---|---|---|---|---|---|---|---|---|---|---|---|
| | | | | | | )oxan-2-yl]oxychromen-4-one | | | | | |
| Pyroglutamylleucine | 8.8 | C11H18N2O4 | [M + H]+ | Dipeptides | pos | Pedunculosumoside G | 14.2 | C22H22O12 | [M - H]- | Flavonoid O-glycosides | neg |
| 2,3-di(dodec-9-enoyloxy)propyl dodec-9-enoate | 35.3 | C39H68O6 | [M + H]+ | Triacylglycerols | pos | Puerol B | 17.9 | C18H16O5 | [M - H]- | Methoxyphenols | neg |
| isoleucylglutamine | 1.5 | C11H21N3O4 | [M + Na]+ | Dipeptides | pos | Daizein | 25.3 | C15H10O4 | [M - H]- | Flavones | neg |
| Ile-Val-Ser | 2.6 | C14H27N3O5 | [M + H]+ | Peptides | pos | BWHPKBOLJFNCPW-YJNULWIISA-N | 28.1 | C21H22O6 | [M - H]- | Curcuminoids | neg |
| [3,4-dihydroxy-5-[6-oxo-2-(2-phenylethyl)-1H-purin-9-yl]oxolan-2-yl]methyl dihydrogen phosphate | 18.2 | C18H21N4O8P | [M + H]+ | Purine ribonucleoside monophosphates | pos | (1R,3S,4R,5R)-1,3,4-trihydroxy-5-(3,4,5-trimethoxybenzoyl)oxycyclohexane-1-carboxylic acid | 19.9 | C17H22O10 | [M - H]- | Cyclitols and derivatives | neg |
| DVBUHWFEXOSULX-JLXBFWJWSA-N | 33.2 | C20H37NO | [M + H]+ | N-acyl amines | pos | Pyroglutamylalanine | 1.7 | C8H12N2O4 | [M - H]- | Dipeptides | neg |

| | | | | | | | | | | | |
|---|---|---|---|---|---|---|---|---|---|---|---|
| Ile-Leu-Ile | 14.8 | C18H35N3O4 | [M + H]+ | Peptides | pos | 5-(7-hydroxy-4-oxo-4H-chromen-3-yl)-1-benzofuran-2-carboxylate | 19.4 | C18H10O6 | [M - H]- | Isoflavones | neg |
| (2S)-2-[[(2S)-2-[[(2S)-1-[(2S)-2-[[(2S)-2-[[(2S)-2-[[(2S)-2-amino-4-methylpentanoyl]amino]-4-methylpentanoyl]amino]propanoyl]amino]-3-methylbutanoyl]pyrrolidine-2-carbonyl]amino]propanoyl]amino]propanoic acid | 13.6 | C31H55N7O8 | [M + H]+ | Peptides | pos | Puerarol | 31.6 | C25H24O5 | [M - H]- | Coumestans | neg |
| Gln-Leu-Ile | 8 | C17H32N4O5 | [M + H]+ | Peptides | pos | gingerol | 27.6 | C17H26O4 | [M - H]- | Gingerols | neg |
| [(2S,3R,4S)-5-(2-benzyl-6-oxo-1H-purin-9-yl)-3,4-dihydroxyoxolan-2- | 11 | C17H19N4O8P | [M + H]+ | Purine ribonucleoside monophospha | pos | (2S,3R,4R,5R,6S)-2-[(2S,3R,4S,5S,6R)-2-[5,7- | 14.9 | C27H30O13 | [M - H]- | Anthocyanins | neg |

| | | | | | | | | | | | |
|---|---|---|---|---|---|---|---|---|---|---|---|
| yl]methyl dihydrogen phosphate | | | | tes | | dihydroxy-2-(4-hydroxyphenyl)chromenylium-8-yl]-4,5-dihydroxy-6-(hydroxymethyl)oxan-3-yl]oxy-6-methyloxane-3,4,5-triol | | | | | |
| Buddledin C | 32.2 | C15H22O | [M + H]+ | Sesquiterpenoids | pos | 3-hydroxy-3-propan-2-ylpentanedioic acid | 9.9 | C8H14O5 | [M - H]- | Hydroxy fatty acids | neg |
| n-benzyloctadecanamide | 34.8 | C25H43NO | [M + H]+ | N-acyl amines | pos | LRXMMYWDVBYTPO-UHFFFAOYSA-N | 17.7 | C16H16O5 | [M - H]- | Stilbenes | neg |
| 3-ethyl-6-propylpiperazine-2,5-dione | 7.7 | C9H16N2O2 | [M + H]+ | Alpha amino acids and derivatives | pos | 3-[5-[(4-hydroxyphenyl)methyl]-3,6-dioxopiperazin-2-yl]propanamide | 2.6 | C14H17N3O4 | [M - H]- | Alpha amino acids and derivatives | neg |
| 3-[(9E,12E)-octadeca-9,12- | 33.1 | C39H68O4 | [M + H]+ | Lineolic acids and | pos | Sinapaldehyde | 10.1 | C11H12O4 | [M - H]- | Methoxyphenols | neg |

| | | | | | | | | | | | |
|---|---|---|---|---|---|---|---|---|---|---|---|
| dienoyl]oxypropyl (9E,12E)-octadeca-9,12-dienoate | | | | derivatives | | | | | | | |
| dodecyl-tris(2-hydroxyethyl)azanium | 26.7 | C18H39NO3 | [M + H]+ | Cholines | pos | Gln-Leu-Leu | 11.6 | C17H32N4O5 | [M - H]- | Peptides | neg |
| alle | 3.3 | C6H13NO2 | [M + H]+ | Alpha amino acids and derivatives | pos | 3,5-dihydroxy-4-methyl-2-[(2S,3R,4R,5S,6R)-3,4,5-trihydroxy-6-(hydroxymethyl)oxan-2-yl]benzoic acid | 7.7 | C14H18O9 | [M - H]- | Phenolic glycosides | neg |
| 11-[(9Z,12Z,15Z,18Z,21Z)-tetracosa-9,12,15,18,21-pentaenoyl]oxypentadecanoic acid | 34.4 | C39H66O4 | [M + H]+ | Long-chain fatty acids | pos | 7,4'-Di-O-methylvitexin 2''-O-rhamnoside | 12.6 | C29H34O14 | [M - H]- | Flavonoid C-glycosides | neg |
| 5-hydroxy-3-[4-hydroxy-3-(3-methylbut-2-enyl)phenyl]-8,8-dimethylpyrano[2,3 | 31.6 | C25H24O5 | [M + H]+ | Pyranoisoflavonoids | pos | 7-hydroxy-4-methyl-3-(4-methyl-2-oxochromen-7-yl)chromen-2- | 30.5 | C20H14O5 | [M - H]- | Hydroxyisoflavonoids | neg |

| | | | | | | | | | | | |
|---|---|---|---|---|---|---|---|---|---|---|---|
| -h]chromen-4-one | | | | | | one | | | | | |
| 2,2,4-trimethyl-3-[3,7,12,16-tetramethyl-18-(2,6,6-trimethylcyclohexen-1-yl)octadeca-1,3,5,7,9,11,13,15,17-nonaenyl]cyclohex-3-en-1-one | 35.1 | C40H54O | [M + H]+ | Xanthophylls | pos | 2-hydroxy-3-(4-hydroxybutyl)butanedioic acid | 4.8 | C8H14O6 | [M - H]- | Medium-chain hydroxy acids and derivatives | neg |
| 10,15-octadecadiynoic acid | 29.7 | C18H28O2 | [M + H]+ | Long-chain fatty acids | pos | 1-(4-chlorophenyl)-3-[2-hydroxy-4-[(3R,4S,5S,6R)-3,4,5-trihydroxy-6-(hydroxymethyl)oxan-2-yl]oxyphenyl]propane-1,3-dione | 11.1 | C21H21ClO9 | [M - H]- | Flavonoid O-glycosides | neg |
| N-[2-acetamido-9-[(2R,3R,4R,5R)-3-fluoro-4-hydroxy-5-(hydroxymethyl)oxolan-2-yl]purin-6- | 28.3 | C14H17FN6O5 | [M + K]+ | Purine nucleosides | pos | 1,7-bis(3,4-dihydroxyphenyl)hepta-1,4,6-trien-3-one | 26.8 | C19H16O5 | [M - H]- | Linear diarylheptanoids | neg |

| | | | | | | | | | | | |
|---|---|---|---|---|---|---|---|---|---|---|---|
| yl]acetamide | | | | | | | | | | | |
| Glu-boroLeu | 1.5 | C10H21BN2O5 | [M + H]+ | Alpha amino acids and derivatives | pos | IANXOBHKQWFRAH-UHFFFAOYSA-N | 17.8 | C16H16O5 | [M - H]- | o-Hydroxybenzoic acid esters | neg |
| (2S,3R)-2-[[(2S,4R)-1-acetyl-4-hydroxypyrrolidine-2-carbonyl]amino]-4-cyano-3-methylbutanoic acid | 3.9 | C13H19N3O5 | [M + Na]+ | Dipeptides | pos | 3-(carboxymethyl)-3-hydroxypentanedioic acid | 1.5 | C7H10O7 | [M - H]- | Tricarboxylic acids and derivatives | neg |
| 1-(4-hydroxy-3-methoxyphenyl)-7-[3-methoxy-4-(3,7,11-trimethyldodeca-2,6,10-trienoxy)phenyl]hepta-1,6-diene-3,5-dione | 33.2 | C36H44O6 | [M + H]+ | Curcuminoids | pos | Cereusitin A | 19.7 | C25H34N4O6 | [M - H]- | Cyclic peptides | neg |
| n-ethyl-n-methylethanolamine | 29.7 | C5H13NO | [M + H]+ | Quaternary ammonium salts | pos | (2S,4S)-4-cyano-2-[[(2S)-3-hydroxy-2-(phenylmethoxy | 11.2 | C17H21N3O6 | [M - H]- | Dipeptides | neg |

| | | | | | | | | | | | |
|---|---|---|---|---|---|---|---|---|---|---|---|
| | | | | | | carbonylamino) propanoyl]amino]pentanoic acid | | | | | |
| N~1~,N~1~-Dibutyl-N~4~-phenylbenzene-1,4-diamine | 30.1 | C20H28N2 | [M + H]+ | Tertiary alkylarylamines | pos | 2-[2-[(1-acetylpyrrolidine-2-carbonyl)amino]propanoylamino]-4-methylpentanoic acid | 12.3 | C16H27N3O5 | [M - H]- | Peptides | neg |
| 2-[[4-(2,5-dimethylphenyl)-5-[(2,4-dioxo-1H-pyrimidin-6-yl)methyl]-1,2,4-triazol-3-yl]sulfanyl]-N-(5-ethylsulfanyl-1,3,4-thiadiazol-2-yl)acetamide | 19.3 | C21H22N8O3S3 | [M + H]+ | Phenyltriazoles | pos | 5-hydroxy-8-(4-hydroxyphenyl)-2,2-dimethylpyrano[2,3-f]chromen-10-one | 29.1 | C20H16O5 | [M - H]- | Pyranoflavonoids | neg |
| 1-(2,4-dimethoxyphenyl)-3-(3-methoxyphenyl)pro | 31.6 | C18H20O4 | [M + H]+ | Phenylpropanoids and polyketides | pos | (6R,7R)-7-(4-carboxybutanoylamino)-3-methylidene-8- | 5 | C13H16N2O6S | [M - H]- | Alpha amino acids and derivatives | neg |

| | | | | | | | | | | | |
|---|---|---|---|---|---|---|---|---|---|---|---|
| pan-1-one | | | | | | oxo-5-thia-1-azabicyclo[4.2.0]octane-2-carboxylic acid | | | | | |
| Nocardiamide B | 24.4 | C35H56N6O7 | [M + H]+ | Cyclic peptides | pos | Garbanzol | 14.6 | C15H12O5 | [M - H]- | Flavanonols | neg |
| (2R)-2-(2-acetamidopropanoylamino)-3-(1H-imidazol-5-yl)propanoic acid | 6.4 | C11H16N4O4 | [M + H]+ | Dipeptides | pos | 1-(4-amino-1,2,5-oxadiazol-3-yl)-5-(3-nitrophenyl)-N'-{(E)-[5-(3-nitrophenyl)furan-2-yl]methylidene}-1H-1,2,3-triazole-4-carbohydrazide | 11.1 | C22H14N10O7 | [M - H]- | Phenyltriazoles | neg |
| Isoallylbenzene | 31.2 | C9H10 | [M + H]+ | Styrenes | pos | (4-acetyl-2-methoxyphenyl)(E)-3-(3,4,5-trimethoxyphenyl)prop-2-enoate | 26.7 | C21H22O7 | [M - H]- | Aryl ketones | neg |
| 1,3-dimethyl-7-phenylpurino[7,8-b][1,2,4]triazine-2,4- | 24.5 | C15H12N6O2 | [M + K]+ | Xanthines | pos | Epiguibourtinidol-4alpha-ol | 17.3 | C15H14O5 | [M - H]- | Leucoanthocyanidins | neg |

| | | | | | | | | | | | |
|---|---|---|---|---|---|---|---|---|---|---|---|
| dione | | | | | | | | | | | |
| (S)-2-amino-N1-((S)-1-oxo-3-phenylpropan-2-yl)succinamide | 5.2 | C13H17N3O3 | [M + H]+ | Alpha amino acids and derivatives | pos | (1E,4Z,6E)-7-(3,4-dihydroxyphenyl)-5-hydroxy-1-(4-hydroxy-3-methoxyphenyl)hepta-1,4,6-trien-3-one | 26.7 | C20H18O6 | [M - H]- | Linear diarylheptanoids | neg |
| 1-(4-methylphenyl)sulfinyldecan-2-one | 34.1 | C17H26O2S | [M + H]+ | Phenyl sulfoxides | pos | Val-Pro-Asp | 5.8 | C14H23N3O6 | [M - H]- | Peptides | neg |
| 4-piperidin-1-ylpyridine-2-carboximidamide | 23.4 | C11H16N4 | [M + Na]+ | Tertiary alkylarylamines | pos | 2-hydroxy-3-[3-(2-hydroxyphenyl)prop-2-enoyloxy]butanedioic acid | 10.1 | C13H12O8 | [M - H]- | Coumaric acid esters | neg |
| 2-[[5-amino-1-[2-(2-amino-3-hydroxypropanoyl)hydrazinyl]-1,5-dioxopentan-2-yl]carbamoylamino]-3- | 5.8 | C13H24N6O8 | [M + H]+ | Alpha amino acids and derivatives | pos | L-Tyrosyl-L-alanyl-L-alanyl-L-phenylalanyl-L-leucine | 20.9 | C30H41N5O7 | [M - H]- | Peptides | neg |

| | | | | | | | | | | | |
|---|---|---|---|---|---|---|---|---|---|---|---|
| hydroxybutanoic acid | | | | | | | | | | | |
| Calebin-A | 26.9 | C21H20O7 | [M + H]+ | Coumaric acids and derivatives | pos | (4-ethenyl-2-hydroxyphenyl) 3-(4-hydroxy-3-methoxyphenyl) prop-2-enoate | 24.4 | C18H16O5 | [M - H]- | Coumaric acids and derivatives | neg |
| Cyclosquamosin D | 22.6 | C41H56N8O11 | [M + H]+ | Cyclic peptides | pos | WRBJZNAPDVBCQM-UHFFFAOYSA-N | 27.8 | C11H8O3 | [M - H]- | Naphthols and derivatives | neg |
| decane-1,4,6-triol | 22.1 | C10H22O3 | [M + Na]+ | Fatty alcohols | pos | [5-[5-[(E)-3-aminoprop-2-enyl]-2,4-dioxopyrimidin-1-yl]-3-[[5-[5-[(E)-3-aminoprop-2-enyl]-2,4-dioxopyrimidin-1-yl]-3-hydroxyoxolan-2-yl]methoxy-hydroxyphosphoryl]oxyoxolan- | 7.5 | C25H36N6O15P2 | [M - H]- | Pyrimidine deoxyribonucleoside bisphosphates | neg |

| | | | | | | | | | | | |
|---|---|---|---|---|---|---|---|---|---|---|---|
| | | | | | | 2-yl]methyl methyl hydrogen phosphate | | | | | |
| [(2S)-3-hydroxy-2-[9-(3-methyl-5-pentylfuran-2-yl)nonanoyloxy]propyl] 9-(3-methyl-5-pentylfuran-2-yl)nonanoate | 32.6 | C41H68O7 | [M + H]+ | Furanoid fatty acids | pos | 3-hydroxy-2-oxobut-3-enoic acid | 1.1 | C4H4O4 | [M - H]- | Short-chain keto acids and derivatives | neg |
| (2S)-N-(3-{(2S)-2-[N-(tert-Butyl)carbamoyl]pyrrolidinyl}(1S,2S)-2-hydroxy-3-oxo-1-benzylpropyl)-2-{(2S)-2-[(phenylmethoxy)carbonylamino]propanoylamino}-3-methylbutanamide | 23.1 | C35H49N5O7 | [M + H]+ | Hybrid peptides | pos | 2-[[2-(4-acetamidoanilino)-2-oxoacetyl]amino]butanedioic acid | 2.3 | C14H15N3O7 | [M - H]- | Peptides | neg |
| (5S)-4,5-dimethylcyclohex-3-en-1-one | 32.2 | C8H12O | [M + H]+ | Cyclic ketones | pos | (4-formyl-2-methoxyphenyl) 4-(4-formyl-2- | 28.3 | C24H20O8 | [M - H]- | Depsides and depsidones | neg |

| | | | | | | | | | | | |
|---|---|---|---|---|---|---|---|---|---|---|---|
| | | | | | | methoxyphenoxy)-3-methoxybenzoate | | | | | |
| (2S)-2-[[(2S)-2-[[(2S)-1-[(2S)-2-[[(2S,3S)-2-[[(2S)-1-[(2S)-1-(4-aminobutanoyl)pyrrolidine-2-carbonyl]pyrrolidine-2-carbonyl]amino]-3-methylpentanoyl]amino]-3-methylbutanoyl]-2,5-dihydropyrrole-2-carbonyl]amino]-3-(4-hydroxyphenyl)propanoyl]amino]-3-phenylpropanoic acid | 21.7 | C48H66N8O10 | [M + Na]+ | Hybrid peptides | pos | tert-butyl N-[(2S)-5-amino-1-[[(2S)-1-hydroxy-3-[(2-methylpropan-2-yl)oxycarbonylamino]propan-2-yl]amino]-1-oxopentan-2-yl]carbamate | 3.3 | C18H36N4O6 | [M - H]- | Alpha amino acids and derivatives | neg |
| 6-[(3-Furylmethyl)amino | 7.5 | C31H26N4O11 | [M + H]+ | Indolocarbazoles | pos | (3,4-dihydroxyphen | 28.3 | C23H19NO9 | [M - H]- | Hippuric acids and | neg |

| | | | | | | | | | | | |
|---|---|---|---|---|---|---|---|---|---|---|---|
| ]-2,10-dihydroxy-13-(beta-D-glucopyranosyl)-12,13-dihydro-5H-indolo[2,3-a]pyrrolo[3,4-c]carbazole-5,7(6H)-dione | | | | | | yl)methyl (Z)-2-[(3,4-dihydroxybenzoyl)amino]-3-(3,4-dihydroxyphenyl)prop-2-enoate | | | | derivatives | |
| 3-acetylsulfanyl-2-(12-nitrooxydodecanoylamino)propanoic acid | 33.5 | C17H30N2O7S | [M + H]+ | Alpha amino acids and derivatives | pos | (2S)-2-[[(2S)-4-amino-2-[[9-[[(2S,3R)-1-[[(2S)-1-amino-1-oxo-3-phenylpropan-2-yl]amino]-3-methyl-1-oxopentan-2-yl]amino]-9-oxononanoyl]amino]-4-oxobutanoyl]amino]-3-cyclohexylpropanoic acid | 19.7 | C37H58N6O8 | [M + H]+ | Dipeptides | pos |
| 2-chloro-N- | 19.3 | C9H16Cl2 | [M + Na]+ | Chloroacetam | pos | (2S,3R)-2-[[(2S)- | 18.7 | C17H25N5O7 | [M + H]+ | Dipeptides | pos |

| | | | | | | | | | | | |
|---|---|---|---|---|---|---|---|---|---|---|---|
| [(2R,4S)-4-[(2-chloroacetyl)amino]pentan-2-yl]acetamide | | $N_2O_2$ | | ides | | 5-(diaminomethylideneamino)-2-[(3,5-dihydroxybenzoyl)amino]pentanoyl]amino]-3-hydroxybutanoic acid | | | | | |
| 7-[(2E)-3,7-dimethylocta-2,6-dienoxy]-6-[(2E)-3,7-dimethylocta-2,6-dienyl]-5-hydroxy-2-phenylchromen-4-one | 32.9 | $C_{35}H_{42}O_4$ | [M + H]+ | Flavones | pos | 2-(N-acetyl-3-chloroanilino)-N-(5-chloro-2-methylphenyl)acetamide | 24.4 | $C_{17}H_{16}Cl_2N_2O_2$ | [M + H]+ | Haloacetanilides | pos |
| Pro-Thr-Pro | 6.7 | $C_{14}H_{23}N_3O_5$ | [M + Na]+ | Peptides | pos | N-(2,6-dimethylpyrimidin-4-yl)acetamide | 32.1 | $C_8H_{11}N_3O$ | [M + H]+ | N-acetylarylamines | pos |
| 6-hydroxy-N-[12-(6-hydroxyhexanoylamino)dodecyl]hexa | 34.1 | $C_{24}H_{48}N_2O_4$ | [M + H]+ | N-acyl amines | pos | (3S)-3-[[(2S)-2-[[2-[[(2S)-2-acetamido-5-amino-5- | 24 | $C_{42}H_{68}N_{12}O_{15}$ | [M + H]+ | Polypeptides | pos |

| | | | | | | | | | | | |
|---|---|---|---|---|---|---|---|---|---|---|---|
| namide | | | | | | oxopentanoyl]amino]acetyl]amino]propanoyl]amino]-4-[[(2S,3R)-1-[(2S)-2-[(2S)-2-[[(2S)-1-[[2-[[(2S)-1-amino-3-methyl-1-oxobutan-2-yl]amino]-2-oxoethyl]amino]-3-methyl-1-oxobutan-2-yl]carbamoyl]pyrrolidine-1-carbonyl]pyrrolidin-1-yl]-3-hydroxy-1-oxobutan-2-yl]amino]-4-oxobutanoic acid | | | | | |
| 2-amino-5-(diaminomethylideneamino)-N-[2-[2- | 16.8 | C10H24N6O5 | [M + H]+ | Alpha amino acids and derivatives | pos | N-[(2S,3S,5R)-5-(6-benzamidopurin | 14.2 | C27H31N8O10P | [M + H]+ | Purine nucleosides | pos |

| | | | | | | | | | | | |
|---|---|---|---|---|---|---|---|---|---|---|---|
| (dihydroxyamino)oxyethoxy]ethyl]pentanamide | | | | | | -9-yl)-2-(hydroxymethyl)oxolan-3-yl]-[[(2R,3S,5R)-3-hydroxy-5-(5-methyl-2,4-dioxopyrimidin-1-yl)oxolan-2-yl]methoxy]phosphonamidic acid | | | | | |
| (2S)-2-[[(2S)-1-[(2S)-1-[(2S)-2-aminopropanoyl]pyrrolidine-2-carbonyl]pyrrolidine-2-carbonyl]amino]-3-hydroxypropanoic acid | 8.1 | C16H26N4O6 | [M + Na]+ | Dipeptides | pos | (4S,14S)-4,14-bis(1H-imidazol-5-ylmethyl)-3,6,9,12,15,21-hexazabicyclo[15.3.1]henicosa-1(21),17,19-triene-2,5,13,16-tetrone | 19.6 | C23H28N10O4 | [M + H]+ | Macrolactams | pos |
| (2R)-2-acetamido-N-[(2S)-1-amino-3-(1H-indol-3-yl)-1-oxopropan-2-yl]-3- | 19.4 | C18H24N4O3 | [M + Na]+ | Dipeptides | pos | [1-[[4-[[3-chloro-1-(3-hydroxypropyl)-2- | 24.4 | C26H33ClF3N8O6P | [M + H]+ | Alpha amino acids and derivatives | pos |

| | | | | | | | | | | | |
|---|---|---|---|---|---|---|---|---|---|---|---|
| methylbutanamide | | | | | | (methylcarbamoyl)-1-(1H-pyrazol-4-yl)-2,6-dihydropyridin-1-ium-3-yl]amino]-5-(trifluoromethyl)pyrimidin-2-yl]amino]-2-methoxy-4-methylcyclohexa-2,4-dien-1-yl]phosphonic acid | | | | | |
| o-aminostyrene | 2.7 | C8H9N | [M + H]+ | Styrenes | pos | N-[4-[5-[(2R)-2-amino-1-phenylpropan-2-yl]-1,3,4-oxadiazol-2-yl]-6-[2-methoxyethyl-[[(1R,2R)-2-methylcyclopropyl]methyl]ami | 30.3 | C28H40N6O4S | [M + Na]+ | Amphetamines and derivatives | pos |

| | | | | | | | | | | | |
|---|---|---|---|---|---|---|---|---|---|---|---|
| | | | | | | no]pyridin-2-yl]-N-methylpropane-2-sulfonamide | | | | | |
| [(3S)-1,3-diamino-2-oxo-4-[(2-phenylacetyl)amino]piperidin-3-yl]-oxophosphanium | 24.5 | C13H17N4O3P | [M + Na]+ | Alpha amino acids and derivatives | pos | tert-butyl N-[1-[(2S)-2-[[1-[[2-[[2-(dimethylamino)-2-oxo-1-phenylethyl]amino]-2-oxoethyl]amino]-5-methyl-1,2-dioxohexan-3-yl]carbamoyl]-4,4-dimethylpyrrolidin-1-yl]-3,3-dimethyl-1-oxobutan-2-yl]carbamate | 19.2 | C37H58N6O8 | [M + H]+ | Dipeptides | pos |
| (2R)-2-acetamido-3-[(2S)-2-acetamido-3-(3-phenylprop-2-enoylsulfanyl)prop | 12.9 | C19H22N2O6S2 | [M + H]+ | Alpha amino acids and derivatives | pos | (2S)-2-[[2-[2-[2-[[2-(2-propoxyethoxy)acetyl]amino]et | 8.6 | C18H34N2O8 | [M + H]+ | Alpha amino acids and derivatives | pos |

| | | | | | | | | | | | |
|---|---|---|---|---|---|---|---|---|---|---|---|
| anoyl]sulfanylpropanoic acid | | | | | | hoxy]ethoxy]acetyl]amino]pentanoic acid | | | | | |
| Protodestruxin | 22 | C28H47N5O7 | [M + H]+ | Cyclic depsipeptides | pos | N-(4-chloro-2-methylphenyl)-2,2-dimethyl-3-(4-methylpiperidin-1-yl)-3-oxopropanamide | 32.7 | C18H25ClN2O2 | [M + H]+ | N-acylpiperidines | pos |
| (2R,5S)-N-[(4S,7R)-11-amino-7-carbamoyl-1-(diaminomethylideneamino)-5-oxoundecan-4-yl]-8-(diaminomethylideneamino)-5-methyl-2-(2-methylpropyl)-4-oxooctanamide | 35.4 | C27H53N9O4 | [M + H]+ | N-acyl amines | pos | (1-fluoro-3-purin-1-ylpropan-2-yl)oxymethylphosphonic acid | 25.9 | C9H12FN4O4P | [M + H]+ | Purines and purine derivatives | pos |
| Pterulamide V | 17 | C38H64N6O6 | [M + H]+ | Peptides | pos | 9-(4-acetylphenoxy)nonan-2-one | 31.3 | C17H24O3 | [M + H]+ | Aryl ketones | pos |

| | | | | | | | | | | | |
|---|---|---|---|---|---|---|---|---|---|---|---|
| (S)-6-(4-chlorobenzylamino)-2-(4-fluoro-3,5-dimethylbenzyl)-N-hydroxyhexanamide | 33.8 | C22H28ClFN2O2 | [M + H]+ | Phenylmethylamines | pos | (2S)-2-[[(2S)-2-amino-4-[2-(2-aminoethylamino)ethylamino]-4-oxobutanoyl]amino]-4-[2-(2-hydroxyethoxy)ethylamino]-4-oxobutanoic acid | 13.7 | C16H32N6O7 | [M + H]+ | Dipeptides | pos |
| 10,11-dimethyl-3-(2-methylpropyl)-13,16-di(propan-2-yl)-4-oxa-1,8,11,14,17-pentazabicyclo[17.3.0]docosane-2,5,9,12,15,18-hexone | 23.7 | C28H47N5O7 | [M + H]+ | Cyclic depsipeptides | pos | 8-(4-methoxybenzoyl)oxyoctyl 4-methoxybenzoate | 28.7 | C24H30O6 | [M + H]+ | P-methoxybenzoic acids and derivatives | pos |

**Table S6** 59 lipid metabolites identified by lipidomics analysis.

| m/z | Metabolite | Molecular formula | model VS ND | model vs HSD (10 μL/mL) |
|---|---|---|---|---|
| | | | logFC | |
| 677.4848 | PE(14:1(9Z)/16:1(9Z)) | C35 H66 N O8 P | -1.13615 | -1.25794 |
| 716.5606 | PE(O-18:0/17:2(9Z,12Z)) | C40 H78 N O7 P | -0.85705 | -1.11821 |
| 979.7217 | PI(22:0/22:0) | C53 H103 O13 P | -0.69236 | -0.68242 |
| 742.6376 | DG(22:3(10Z,13Z,16Z)/22:3(10Z,13Z,16Z)/0:0) | C47 H80 O5 | -0.63697 | -0.6957 |
| 582.5452 | Cer(d14:1(4E)/22:0(2OH)) | C36 H71 N O4 | -0.6045 | -0.70129 |
| 750.5518 | GlcCer(d15:2(4E,6E)/20:0(2OH)) | C41 H77 N O9 | -0.59794 | -0.59241 |
| 869.7177 | PC(P-20:0/21:0) | C49 H99 N O7 P | -0.58065 | -0.7246 |
| 790.692 | SM(d18:0/22:0) | C45 H94 N2 O6 P | -0.54759 | -0.67147 |
| 827.6002 | PC(18:0/20:4(5Z,8Z,10E,14Z)(12OH[S])) | C46 H85 N O9 P | -0.52759 | -0.56316 |
| 759.6384 | SM(d18:1/20:0) | C43 H88 N2 O6 P | -0.52235 | -0.54866 |
| 562.4815 | 35-aminobacteriohopane-31,32,33,34-tetrol | C35 H63 N O4 | -0.50279 | -0.55216 |
| 700.5709 | GlcCer(d18:1/16:0) | C40 H77 N O8 | -0.45541 | -0.48063 |
| 772.6115 | PC(O-15:0/20:4(5Z,8Z,11Z,14Z)) | C43 H81 N O7 P | -0.45317 | -0.35041 |

| | | | | |
|---|---|---|---|---|
| 707.4957 | PA(O-18:0/18:3(6Z,9Z,12Z)) | C39 H73 O7 P | -0.4476 | -0.69982 |
| 769.5735 | PA(19:1(9Z)/22:2(13Z,16Z)) | C44 H81 O8 P | -0.44261 | -0.51723 |
| 731.5804 | GlcCer(d14:2(4E,6E)/20:0(2OH)) | C40 H75 N O9 | -0.43173 | -0.56967 |
| 649.48 | (2-methoxy-12-methyloctadec-17-en-5-ynoyl) 2-methoxy-12-methyloctadec-17-en-5-ynoate | C40 H66 O5 | -0.41327 | -0.50498 |
| 711.494 | DG(20:5(5Z,8Z,11Z,14Z,17Z)/22:5(7Z,10Z,13Z,16Z,19Z)/0:0) | C45 H68 O5 | -0.40701 | -0.50837 |
| 784.586 | PC(16:0/20:3(11E,14E,17E))[U] | C44 H83 N O8 P | -0.40556 | -0.41518 |
| 726.5385 | PE(16:0/P-18:0) | C39 H78 N O7 P | -0.40401 | -0.43256 |
| 690.5434 | PC(14:0/P-16:0) | C38 H77 N O7 P | -0.38505 | -0.5057 |
| 918.6033 | PI(17:0/22:4(7Z,10Z,13Z,16Z)) | C48 H85 O13 P | -0.37566 | -0.61362 |
| 825.6542 | PC(20:0/P-18:0) | C46 H93 N O7 P | -0.36898 | -0.59977 |
| 840.5739 | PS(18:0/22:4(7Z,10Z,13Z,16Z)) | C46 H82 N O10 P | -0.34251 | -0.23218 |
| 719.5675 | PE-Cer(d15:1(4E)/22:0(2OH)) | C39 H79 N2 O7 P | -0.29265 | -0.44556 |
| 972.7341 | Galbeta1-4Glcbeta-Cer(d18:1/24:1(15Z)) | C54 H101 N O13 | -0.2618 | -0.1774 |
| 536.5022 | Cer(d18:2/16:0) | C34 H65 N O3 | -0.25559 | -0.40498 |
| 741.531 | PC(20:4(5Z,8Z,11Z,14Z)/13:0) | C41 H75 N O8 P | -0.23595 | -0.27008 |

| | | | | |
|---|---|---|---|---|
| 826.6703 | PC(22:1(13Z)/P-18:1(11Z)) | C48 H93 N O7 P | -0.23545 | -0.40411 |
| 814.6339 | PC(14:1(9Z)/24:1(15Z)) | C46 H89 N O8 P | -0.22953 | -0.23925 |
| 780.5902 | PE(P-20:0/20:4(5Z,8Z,11Z,14Z)) | C45 H82 N O7 P | -0.2055 | -0.26236 |
| 493.2918 | Minabeolide-8 | C29 H42 O5 | -0.20456 | -0.34715 |
| 925.5183 | CL(1'-[18:2(9Z,12Z)/0:0],3'-[18:2(9Z,12Z)/0:0]) | C45 H82 O15 P2 | -0.19435 | -0.20122 |
| 890.6573 | Galbeta1-4Glcbeta-Cer(d18:1/18:0) | C48 H91 N O13 | -0.19297 | -0.24606 |
| 713.511 | PA(21:0/14:0) | C38 H75 O8 P | -0.19293 | -0.3058 |
| 842.6648 | PC(20:0/20:2(11Z,14Z)) | C48 H93 N O8 P | -0.19091 | -0.23567 |
| 648.4953 | PE(14:0/P-16:0) | C35 H70 N O7 P | -0.18197 | -0.19783 |
| 849.6321 | PS(17:0/22:1(11Z)) | C45 H86 N O10 P | -0.17428 | -0.20205 |
| 668.4656 | PE(14:1(9Z)/P-16:0) | C35 H68 N O7 P | -0.1681 | -0.48492 |
| 856.7125 | 3-decaprenyl-4,5-dihydroxybenzoic acid | C57 H86 O4 | -0.13451 | -0.16821 |
| 816.613 | PS(P-20:0/19:1(9Z)) | C45 H86 N O9 P | -0.1169 | -0.167 |
| 785.5789 | PE(16:1(9Z)/20:3(8Z,11Z,14Z)) | C43 H78 N O8 P | 0.098591 | 0.095914 |
| 717.552 | PE-Cer(d15:2(4E,6E)/22:0(2OH)) | C39 H77 N2 O7 P | 0.171107 | 0.123883 |
| 702.5449 | PC(P-16:0/15:1(9Z)) | C39 H77 N O7 P | 0.187942 | 0.128465 |

| | | | | |
|---|---|---|---|---|
| 254.2113 | 4E,7Z,10Z-Tridecatrienyl acetate | C15 H24 O2 | 0.204894 | 0.21448 |
| 397.2716 | PA(O-16:0/0:0) | C19 H41 O6 P | 0.208475 | 0.225798 |
| 436.3403 | Dichotellate A | C26 H42 O4 | 0.220799 | 0.346273 |
| 656.4497 | PG(14:0/12:0) | C32 H63 O10 P | 0.23073 | 0.420018 |
| 878.6954 | PC(O-22:2(13Z,16Z)/22:3(10Z,13Z,16Z)) | C52 H97 N O7 P | 0.236111 | 0.238842 |
| 730.5762 | PC(15:0/P-18:1(11Z)) | C41 H81 N O7 P | 0.246323 | 0.197915 |
| 664.4907 | PE(12:0/18:0) | C35 H70 N O8 P | 0.258179 | 0.327344 |
| 965.754 | TG(17:1(9Z)/20:5(5Z,8Z,11Z,14Z,17Z)/22:3(10Z,13Z,16Z)) | C62 H102 O6 | 0.288675 | 0.341331 |
| 974.7478 | Galbeta1-4Glcbeta-Cer(d18:1/24:0) | C54 H103 N O13 | 0.302183 | 0.304982 |
| 691.5297 | PA(16:0/19:0) | C38 H75 O8 P | 0.318967 | 0.274953 |
| 776.5605 | PE(22:5(4Z,7Z,10Z,13Z,16Z)/P-18:1(11Z)) | C45 H78 N O7 P | 0.337556 | 0.261979 |
| 707.5961 | SM(d16:1/17:0) | C38 H78 N2 O6 P | 0.532278 | 0.536869 |
| 728.5609 | PE(O-16:0/20:3(8Z,11Z,14Z)) | C41 H78 N O7 P | 0.544825 | 0.376261 |
| 800.6172 | PE(22:1(11Z)/18:1(9Z)) | C45 H86 N O8 P | 0.574621 | 0.466067 |
| 807.5399 | PI(O-16:0/17:2(9Z,12Z)) | C42 H79 O12 P | 0.634803 | 0.537601 |

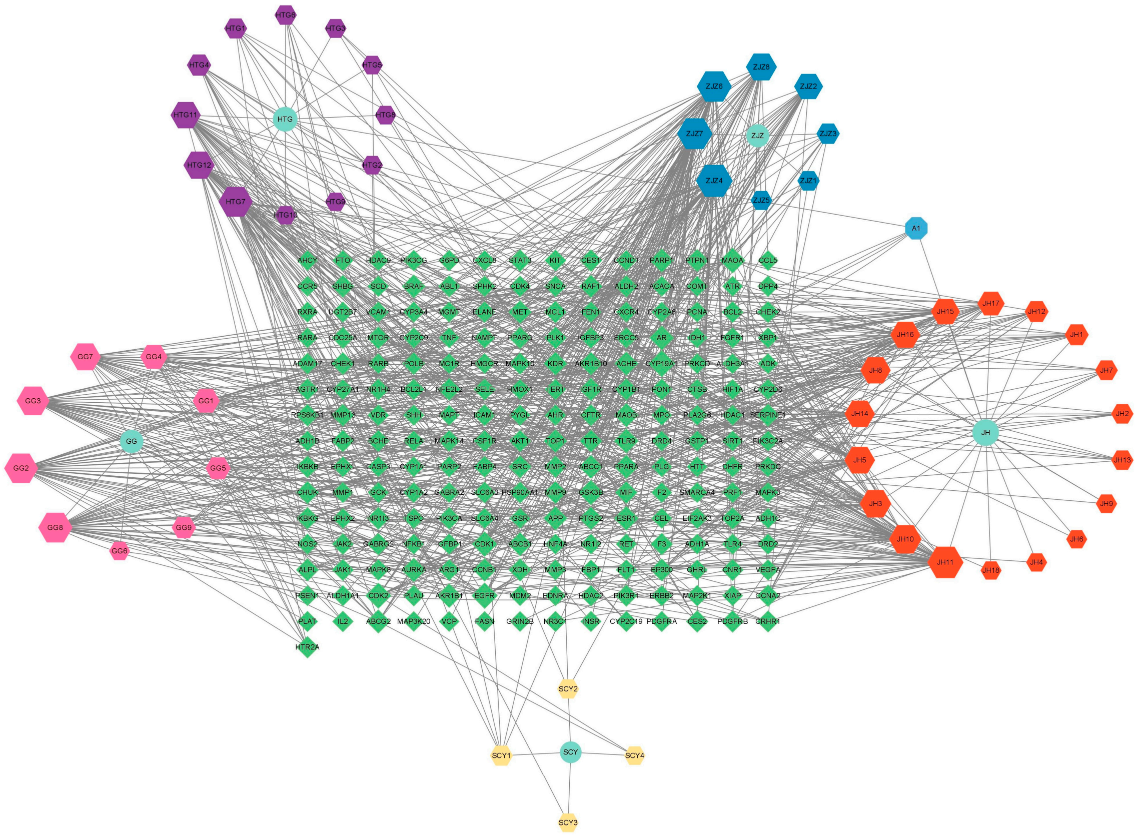

**Figure S1.** A complex target pathway network of 52 main active ingredients. (Green diamonds represent targets, red hexagons represent active ingredients from *Rhizoma Curcumae longae*, purple hexagons represent active ingredients from *Hericium erinaceus*, blue hexagons represent active ingredients from *Hoveniae Dulcis* Semen, pink hexagons represent active ingredients from *Radix Puerariae*, yellow hexagons represent active ingredients from *Camellia oleifera*, and A1 is *Hericium erinaceus* and *Rhizoma Curcumae longae* poses overlapping active constants).

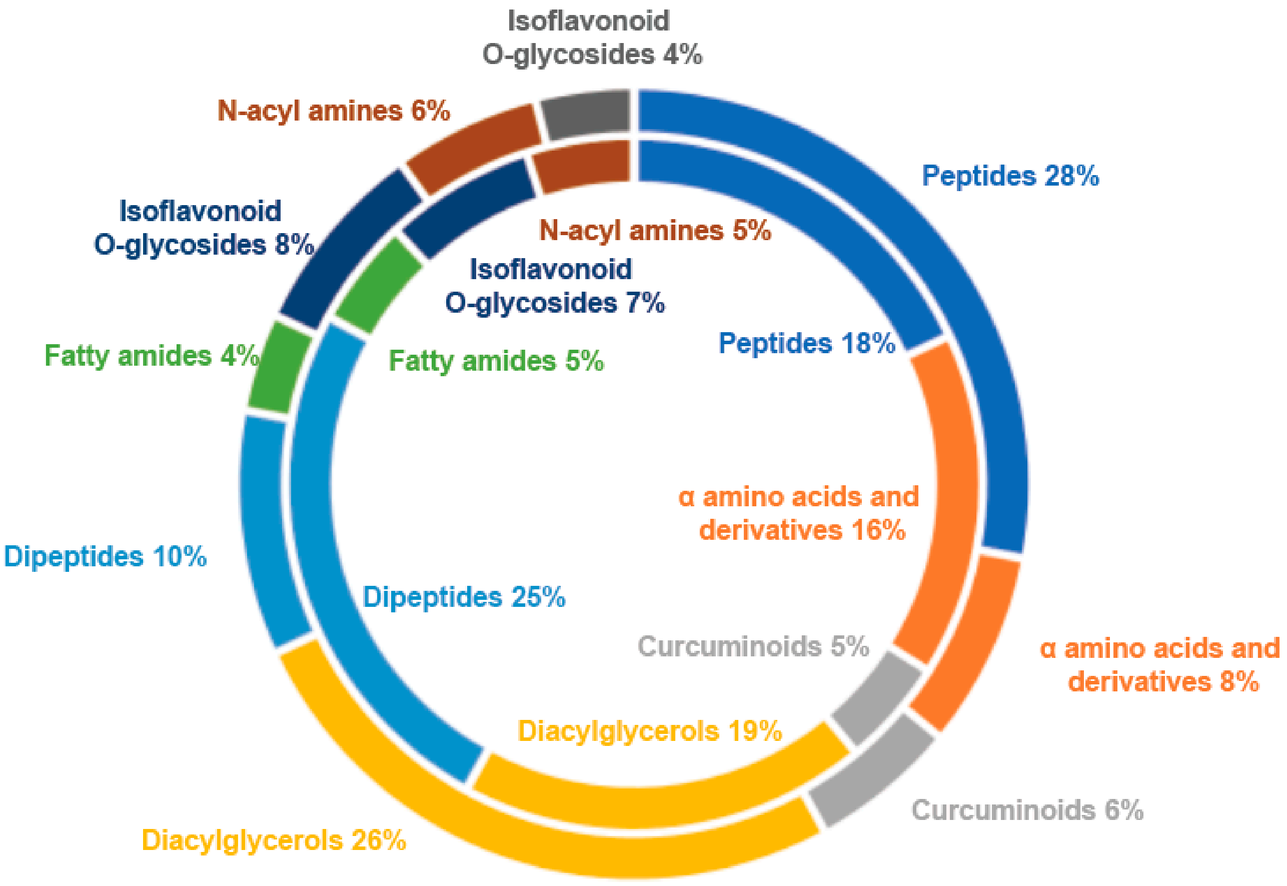


**Figure S2.** Metabolite identification and classification of "HepaSynergy Decoction" The inner circle has a positive spectrum, while the outer circle has a negative spectrum.

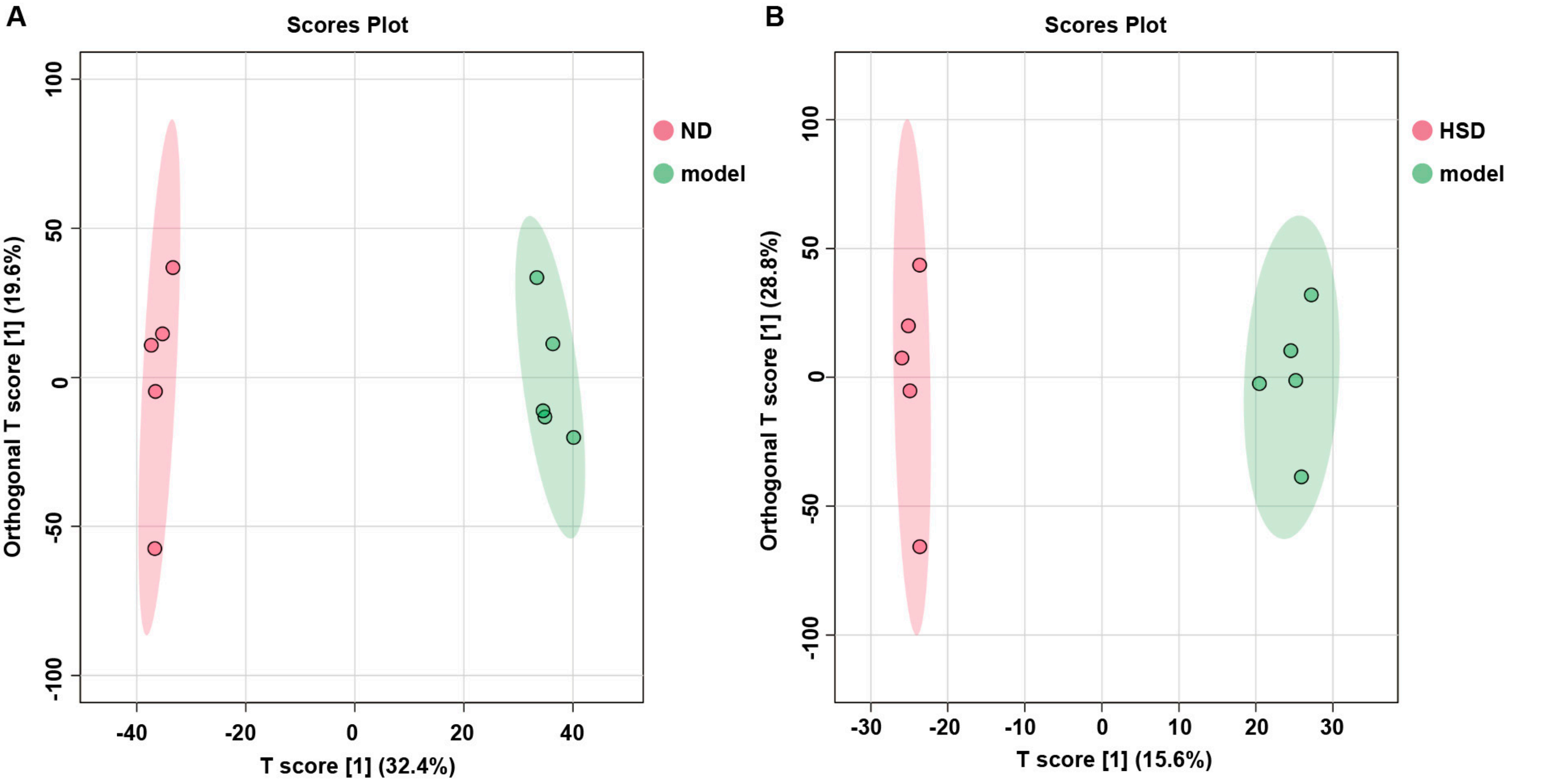


**Figure S3.** (A) PLS-DA of lipids in high-fat induced cells (model) and HepG2 cells (ND); (B) PLS-DA of lipids in high-fat induced cells (model) treated with 10 μL $mL^{-1}$ HepaSynergy Decoction (HSD).
